# Thymidylate synthase accelerates *Men1*-mediated pancreatic tumor progression and reduces survival

**DOI:** 10.1172/jci.insight.147417

**Published:** 2022-10-10

**Authors:** Vinod Vijayakurup, Kyungah Maeng, Hye Seung Lee, Benjamin Meyer, Sandra Burkett, Akbar Nawab, Michael W. Dougherty, Christian Jobin, Iqbal Mahmud, Timothy J. Garrett, Michael Feely, Kyoung Bun Lee, Frederic J. Kaye, Maria V. Guijarro, Maria Zajac-Kaye

**Affiliations:** 1 Department of Anatomy and Cell Biology, University of Florida College of Medicine, Gainesville, Florida, USA.; 2 Department of Pathology, Seoul National University Hospital, Seoul National University College of Medicine, Seoul, South Korea.; 3 Molecular Cytogenetics Core Facility, Center for Cancer Research, National Cancer Institute, NIH, Frederick, Maryland, USA.; 4 Department of Medicine and; 5 Department of Pathology, Immunology and Laboratory Medicine, University of Florida College of Medicine, Gainesville, Florida, USA.

**Keywords:** Oncology, Mouse models, Oncogenes, Tumor suppressors

## Abstract

Clinical studies of cancer patients have shown that overexpression or amplification of thymidylate synthase (TS) correlates with a worse clinical outcome. We previously showed that elevated TS exhibits properties of an oncogene and promotes pancreatic neuroendocrine tumors (PanNETs) with a long latency. To study the causal impact of elevated TS levels in PanNETs, we generated a mouse model with elevated human TS (hTS) and conditional inactivation of the *Men1* gene in pancreatic islet cells (*hTS/Men1^–/–^*). We demonstrated that increased hTS expression was associated with earlier tumor onset and accelerated PanNET development in comparison with control *Men1^–/–^* and *Men1*^+/*Δ*N3-8^ mice. We also observed a decrease in overall survival of *hTS/Men1^+/–^* and *hTS/Men1^–/–^* mice as compared with control mice. We showed that elevated hTS in *Men1*-deleted tumor cells enhanced cell proliferation, deregulated cell cycle kinetics, and was associated with a higher frequency of somatic mutations, DNA damage, and genomic instability. In addition, we analyzed the survival of 88 patients with PanNETs and observed that high TS protein expression independently predicted worse clinical outcomes. In summary, elevated hTS directly participates in promoting PanNET tumorigenesis with reduced survival in *Men1*-mutant background. This work will refocus attention on new strategies to inhibit TS activity for PanNET treatment.

## Introduction

Pancreatic neuroendocrine tumors (PanNETs) are neoplasms arising from specialized islet cells within the pancreas, conferring a median overall survival of 3.6 years based on estimates for tumors diagnosed between 1973 and 2012 (1). A statistically significant increase in the incidence of PanNETs has been reported in past decades (2), coinciding with new insights about molecular and cell signaling pathways underlying tumor progression (3, 4). Recent studies analyzing the genomic landscape of PanNETs show that key genes regulating mTOR signaling, histone modifications, telomere length, and DNA damage are targets for mutations in PanNET tissues (5, 6). Among these aberrations, somatic inactivation of *MEN1*, a tumor suppressor gene that acts as a nuclear scaffold to modulate chromatin structure and selectively regulate gene transcription, is among the most common events in the initiation of PanNETs (5, 7). Mice with heterozygous *Men1* deletion develop a spectrum of endocrine tumors, including pancreatic islet tumors, similar to that observed in human patients carrying germline mutant alleles (8). Transgenic mouse models with conditional deletion of *Men1* in pancreatic islet cells develop PanNETs. However, these mice develop tumors only after a prolonged latency period (9), which suggests that islet tissues must acquire additional sequential mutations to cooperate with *Men1* mutation for progression to PanNET. Recently, mutational inactivation of *Men1* was observed concurrent with loss of phosphatase and tensin homolog, a gene regulating the PKB/Akt pathway in the progression of PanNETs (10). Additional events that could cooperate with *Men1* mutation for the progression of PanNETs are currently unknown, partly because of the lack of animal models to test and validate these cooperative aberrant events.

We recently reported that elevated levels of thymidylate synthase (TS), an enzyme linked to DNA metabolism (11), have a pro-tumorigenic role in PanNETs (12). TS is the sole source of thymidylate production that is required for DNA synthesis (13). Elevated expression level of TS is detected in a wide spectrum of tumors and is consistently associated with poor prognosis in cancer patients (13–19). We have previously shown that ectopic TS can transform rodent cells and induce tumors in nude mice (20). To extend these findings, we developed a transgenic murine model (*CMV-hTS*) that overexpresses human TS (hTS) in pancreatic tissues and showed that elevated TS induces a phenotype resembling PanNET (12). *CMV-hTS* mice develop pancreatic endocrine abnormalities ranging from hyperplasia to adenoma. However, progression of these tumors to islet adenoma occurred only at a modest frequency and required a latency period of 9 to 24 months (12). This modest frequency and long latency suggested that elevated TS levels alone may not be a core tumor-driving event in PanNETs and that TS overexpression may cooperate with other tumor-initiating events. Our recent clinical studies show that overexpression of TS in tumors correlates with worse prognosis in patients with PanNETs (15). To test for gene mutations that could cooperate with elevated TS in PanNET progression, we selected *Men1*, which is highly mutated in PanNET patients (3, 6).

Here, we report a potentially novel PanNET mouse model where TS overexpression cooperates with *Men1* inactivation in pancreatic islet cells. We demonstrated that elevated hTS directly accelerated PanNET progression and reduced survival of a genetically engineered *hTS/Men1^–/–^* mouse model. We also found that elevated levels of TS induced cellular proliferation, transformation, and genomic instability such as DNA damage and chromosomal aberrations in *Men1*-null mouse embryo fibroblast cells. Moreover, we show that reduction of TS levels in a human neuroendocrine tumor cell line downregulates p21^Cip1^ levels and reduces tumor cell proliferation. In addition, we observed TS-induced G_2_/M arrest and provide evidence for TS-induced somatic mutations in *Men1*-deleted islet tissues isolated from our newly developed mouse model. This work will refocus attention on developing new strategies to inhibit TS activity for PanNET treatment.

## Results

### TS overexpression shortens survival of Men1^–/–^ and Men1^+/–^ mice.

**Figure 1:**
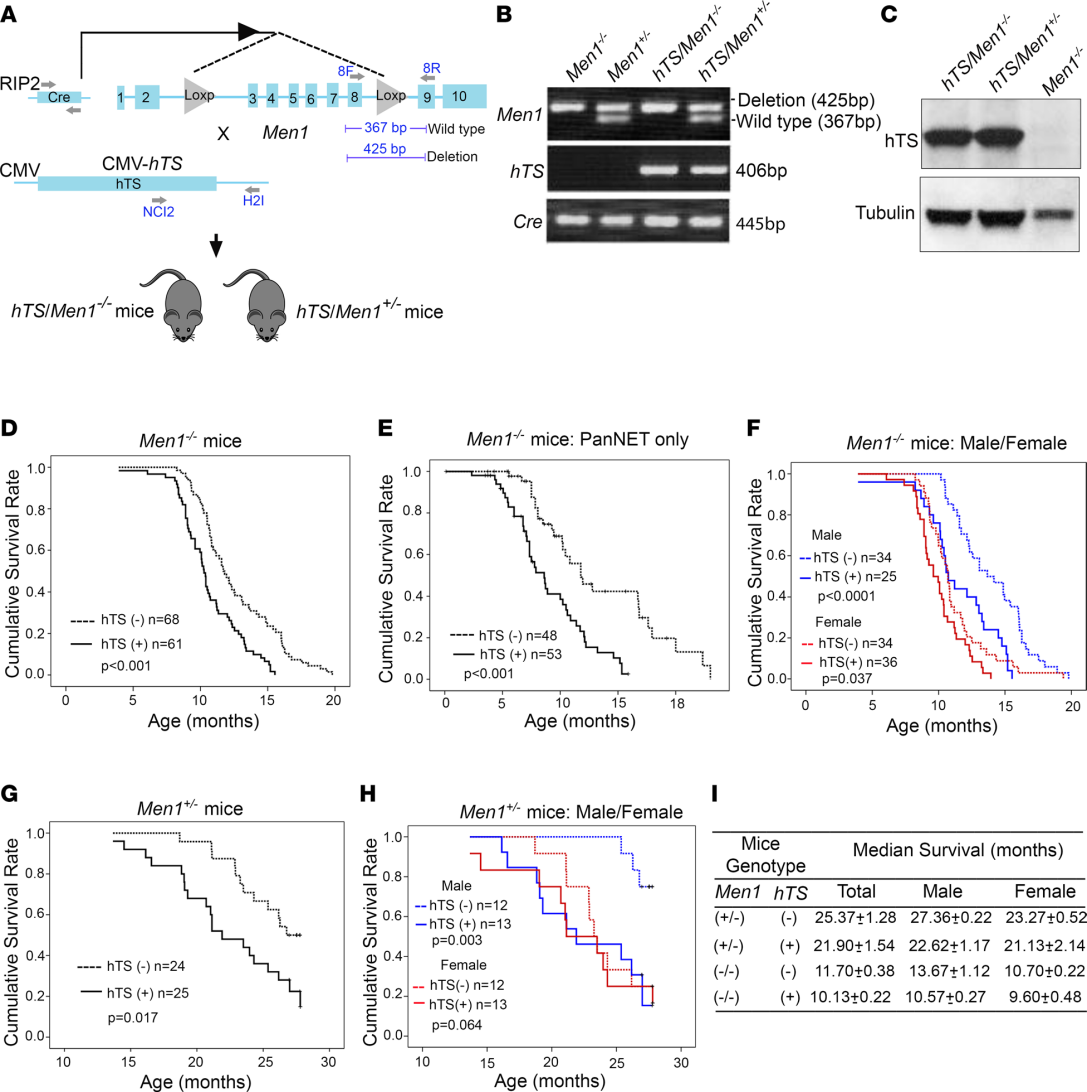
Overexpression of hTS shortens the survival of *Men1^–/–^* and *Men1^+/–^* mice. (**A**) Schematic representation of breeding strategy to generate *hTS/Men1^–/–^* and *hTS/Men1^+/–^* mouse models. Location of forward (F) and reverse (R) primers for the detection of *hTS* transgene, RIP2-*Cre* gene, and identification of *lox* insert in *Men1* gene are shown with arrows. (**B**) Representative picture of PCR analysis of DNA from mouse tail showing heterozygous status of *Men1* gene in *Men1^+/–^* versus *hTS/Men1^+/–^* mice (425 and 367 bp bands), *hTS* (406 bp band), and *Cre* (445 bp band). (**C**) Immunoblot analysis of hTS (37 kDa) in pancreatic tissues isolated from *hTS/Men1^–/–^*, *hTS/Men1^+/–^*, and *Men1^–/–^* mice. Tubulin was used as loading control. (**D**) Kaplan-Meier analysis of overall survival in homozygous *Men1^–/–^* versus *hTS/Men1^–/–^* mice (*P* < 0.001). (**E**) Kaplan-Meier survival plot for *Men1^–/–^* versus *hTS/Men1^–/–^* mice that exclusively developed PanNETs (*P* < 0.001). (**F**) Kaplan-Meier survival plot for male *Men1^–/–^* versus *hTS/Men1^–/–^* mice (*P* < 0.0001) and female *Men1^–/–^* versus *hTS/Men1^–/–^* mice (*P* = 0.037). (**G**) Kaplan-Meier analysis of overall survival in heterozygous *Men1^+/–^* versus *hTS/Men1^+/–^* mice (*P* = 0.017). (**H**) Kaplan-Meier survival plot for male *Men1^+/–^* versus *hTS/Men1^+/–^* mice (*P* = 0.003) and female *Men1^+/–^* versus *hTS/Men1^+/–^* mice (*P* = 0.064). (**I**) Median survival of mice based on sex and genotype from **D** and **F**–**H**.

We previously demonstrated that transgenic mice expressing human TS (hTS) in the pancreas develop islet cell tumors. However, tumor development occurred with a long latency (up to 24 months of age), suggesting that constitutively elevated TS does not act alone but rather requires cooperating genetic events to promote tumorigenesis (12). Therefore, we were interested in studying oncogenes or tumor suppressor genes associated with tumors of the endocrine pancreas as candidates that may cooperate with ectopic TS expression in a murine transgene model. We initially tested whether TS may cooperate with *Men1*, because obligate carriers with familial multiple endocrine neoplasia type 1 syndrome (MEN1 syndrome) develop pancreatic islet cell tumors, and *Men1*-mutant mice develop only pancreatic adenoma after a long latency of 9 months (8). In addition, the *MEN1* gene is the most frequent target for somatic mutations in human PanNETs (5, 6). To determine whether TS overexpression will accelerate the development of PanNETs in *Men1*-mutant mice, we crossed *RIP2-Cre/floxed Men1* mice (*Men1^–/–^* mice) (9) with *CMV-hTS* mice (*hTS* mice) (12) and generated *CMV-hTS/RIP2-Cre/floxed Men1* mice (designated *hTS/Men1^–/–^* and *hTS/Men^+/–^* mice) (Figure 1A). Using PCR analysis, we confirmed the presence of *loxP*-*Men1* DNA fragment (425 bp band) in *Men1^–/–^* mice, while *Men^+/–^* mice were identified by the presence of heterozygous bands as follows: wild-type *Men1*, 367 bp band; and *loxP*-*Men1*, 425 bp band (Figure 1B). The presence of *hTS* in transgenic *hTS/Men1^–/–^* and *hTS/Men^+/–^* mice was confirmed by detection of the 406 bp band (Figure 1B). RIP2-*Cre* was confirmed by the presence of a 445 bp band (Figure 1B), and protein expression of hTS in pancreas of *hTS/Men1^–/–^* and *hTS/Men^+/–^* mice was determined by immunoblot analysis (Figure 1C). In addition, we confirmed by IHC analysis that tumors developed in *Men1^–/–^* and *hTS/Men1^–/–^* mice were of neuroendocrine origin, since we detected synaptophysin and chromogranin A in tumor tissues (Supplemental Figure 1; supplemental material available online with this article; https://doi.org/10.1172/jci.insight.147417DS1).

We observed that hTS overexpression accelerated PanNET progression and that high levels of TS significantly shortened survival of homozygous *Men1*-null (*Men1^–/–^*) and heterozygous *Men^+/–^* mice (Figure 1, D–I). A large cohort of *hTS/Men1^–/–^* (*n* = 61) and *Men1^–/–^* (*n* = 68) as well as *hTS/Men1^+/–^* (*n* = 25) and *Men1^+/–^* (*n* = 24) mice were followed until death or when mice were required to be euthanized owing to symptoms of tumor burden per IACUC guidelines. We observed that *hTS/Men1^–/–^* mice displayed a statistically significant decrease in survival as compared with *Men1^–/–^* mice (*P* < 0.001) (Figure 1, D and I). The median survival time of *hTS/Men1^–/–^* mice (10.13 ± 0.22 months) was 1.5 months shorter than that of *Men1^–/–^* mice (11.70 ± 0.38 months) (Figure 1, D and I). Since a subset of these mice also developed pituitary tumors, we analyzed the survival of mice that only developed PanNETs without the development of pituitary tumors. We found that hTS overexpression significantly shortened survival of the subset of *Men1^–/–^* mice that only developed PanNETs (2.34 months shorter than survival of *Men1^–/–^* mice that developed PanNETs only; *P* < 0.001) (Figure 1E).

Since it has been shown that PanNET clinical outcome is better in men than in women (21), we compared survival differences between *hTS-*positive and *hTS*-negative male versus female mice (Figure 1, F and I). Our data demonstrate that female *Men1^–/–^* mice died earlier than male mice and that overexpression of TS decreased survival in both sexes but to a greater extent in male mice (Figure 1, F and I). We also observed a greater reduction in survival in male *hTS/Men1^–/–^* mice compared with control *Men1^–/–^* male mice. The median survival time of *hTS/Men1^–/–^* male mice was 3.1 months shorter than that of *Men1^–/–^* male mice (10.57 ± 0.27 vs. 13.67 ± 1.12 months, respectively, *P* < 0.0001) (Figure 1, F and I). The difference of median survival time in *hTS/Men1^–/–^* female mice was 1.1 months shorter in comparison with *Men1^–/–^* mice (9.60 ± 0.48 vs. 10.70 ± 0.22 months, *P* = 0.037) (Figure 1, F and I).

In addition, we compared the effect of hTS overexpression on the life span of conditional heterozygote *hTS/Men1^+/–^* versus *Men1^+/–^* mice. We found that TS overexpression reduced survival of *Men1^+/–^* mice by 3.5 months (median survival 21.90 ± 1.54 vs. 25.37 ± 1.28 months, respectively, *P* < 0.017) (Figure 1, G and I). Comparing male and female (*hTS/Men1^+/–^* vs. *Men1^+/–^*) cohorts, we again observed a greater reduction in the survival of male mice. The mean survival time of *hTS/Men1^+/–^* male mice was 5 months shorter than that of male *Men1^+/–^* mice (22.62 ± 1.17 vs. 27.36 ± 0.22 months, respectively, *P* = 0.003) (Figure 1, H and I). There was a difference of 2.1 months in survival of female *hTS/Men1^+/–^* mice as compared with female *Men1^+/–^* mice (21.13 ± 2.14 vs. 23.27 ± 0.52 months, respectively), but the difference was not statistically significant (*P* = 0.064) (Figure 1, H and I). Our data also demonstrate that female *Men1^–/–^* and *Men1^+/–^* mice had reduced survival compared with their male counterparts and that overexpression of TS decreased survival in both sexes but to a greater extent in male mice. A possible explanation may be that female *Men1^–/–^* and *Men1^+/–^* mice died earlier because they developed pituitary tumors more frequently than male mice. This corresponds to the observation that the clinical course of non-functioning pituitary adenoma seems to differ in women and men and the outcome is better in men than in women (21). Our data showed that TS overexpression reduced survival to a greater extent in *Men1^–/–^* and *Men1^+/–^* male over female mice (Figure 1, F and H), suggesting that TS may enhance growth of PanNETs to a greater extent than that of pituitary tumors. Taken together, our results demonstrate that overexpression of hTS reduced survival of both *hTS/Men1^–/–^* and *hTS/Men1^+/–^* as compared with *Men1^–/–^* and *Men1^+/–^* mice. In addition, we observed that male *Men1^–/–^* and *Men1^+/–^* mice lived longer than female mice and the reduction in survival due to TS overexpression was more pronounced in male *Men1^–/–^* and *Men1^+/–^* mice as compared with female mice.

### hTS overexpression increases incidence and accelerates progression of PanNETs in Men1^–/–^ and Men1^+/ΔN3-8^ mice.

**Figure 2:**
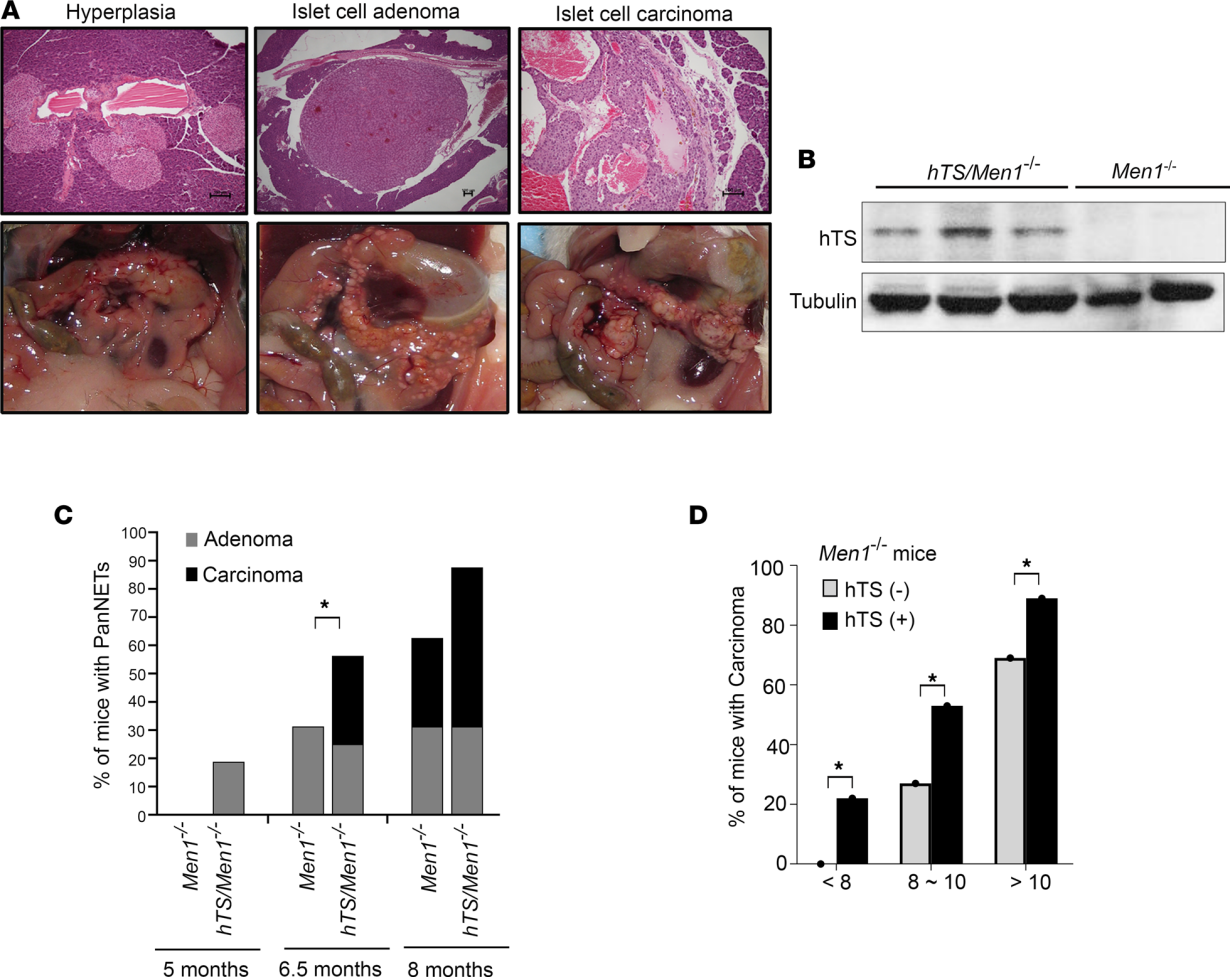
Overexpression of hTS accelerates PanNET progression in mice with conditional *Men1* knockout. (**A**) Representative H&E images and photographs of pancreatic lesions developed in *hTS/Men1^–/–^* mice (scale bars: 100 μm). (**B**) Immunoblot analysis of hTS in carcinoma isolated from *hTS/Men1^–/–^* and *Men1^–/–^* mice. (**C**) Comparison of adenoma and carcinoma incidence in *Men1^–/–^* and *hTS/Men1^–/–^* mice at 5, 6.5, and 8 months (from data shown in Supplemental Table 1). (**D**) Comparison of carcinoma incidence in *Men1^–/–^* and *hTS/Men1^–/–^* mice sacrificed at defined time points combined with mice sacrificed at end point. In **C** and **D**, significance was calculated using χ^2^ test, *P < 0.05.

Since we observed that TS overexpression significantly decreased survival of *Men1^–/–^* mice, we asked whether high levels of hTS will impact the rate of PanNET development. To test this, we euthanized *hTS/Men1^–/–^* and control *Men1^–/–^* mice at 5, 6.5, and 8 months of age (*n* = 16 for each age group). The entire pancreas was collected at each time point, fixed, and stained with H&E for histopathological analysis. Each pancreas was scored by the same masked veterinary pathologist, according to criteria previously described (22), who identified presence of normal or hyperplastic foci, adenoma, or carcinoma. Representative H&E images (Figure 2A, top) and macroscopic photographs (Figure 2A, bottom) demonstrate progression of pancreatic lesions from hyperplasia and adenoma to carcinoma. Multiple islets with diameters greater than 100 μm were found with normal architecture and classified as hyperplasia. Adenoma was scored by detection of islets larger than 100 μm that were associated with prominent vascular dilation and compression of adjacent acinar pancreas. Carcinoma showed capsular invasion and increased cellular pleomorphism (Figure 2A). We also performed TS immunoblotting and confirmed presence of hTS in carcinoma from *hTS/Men1^–/–^* but not *Men1^–/–^* mice (Figure 2B).

Comparison of the histological features of islet lesions between *Men1^–/–^* and *hTS/Men1^–/–^* mice for each age group showed that hTS overexpression was associated with accelerated neuroendocrine tumor development in comparison with *Men1^–/–^* controls (Figure 2C and Supplemental Table 1). We observed that 3 of 16 *hTS/Men1^–/–^* mice (18.8%) developed islet adenomas at 5 months of age while 0 of 16 of *Men1^–/–^* mice developed adenomas at this time point (Supplemental Table 1). As expected, the percentage of hyperplastic lesions decreased at 6.5 and 8 months of age, since these mice developed adenoma in the presence of high TS levels (Supplemental Table 1). For example, the progression to adenoma and carcinoma was pronounced at 6.5 months: 5 of 16 *hTS/Men1^–/–^* mice developed carcinoma (31.3%) as compared with 0 of 16 *Men1^–/–^* mice with carcinoma at this time point (0%). We also observed that 9 of 16 *hTS/Men1^–/–^* mice (56.3%) and 5 of 16 *Men1^–/–^* mice (31.3%) developed carcinoma at 8 months of age (Supplemental Table 1). These data suggest that TS overexpression in *Men1*-null background increased the incidence of adenoma (Figure 2C, gray columns) and carcinoma (Figure 2C, black columns) as early as 5 and 6.5 months of age, respectively, in comparison with *Men1^–/–^* mice (Figure 2C).

In addition to analyzing pancreatic lesions in *hTS/Men1^–/–^* and *Men1^–/–^* mice at defined time points (Supplemental Table 1), we also analyzed mice that were euthanized at end point. Combining data from all mice (Figure 1D and Supplemental Table 1), we compared the incidence of pancreatic islet carcinoma in *hTS/Men1^–/–^* versus age-matched control *Men1*^–/–^ mice (Figure 2D and Supplemental Table 2). Before 8 months of age, 21.7% of *hTS/Men1^–/–^* (*n* = 23) mice had carcinomas as compared with 0% carcinoma in *Men1^–/–^* mice (*n* = 20) (*P* < 0.05). At 8–10 months of age, 53.3% of *hTS/Men1^–/–^* mice developed carcinomas whereas only 26.7% of *Men1^–/–^* mice had carcinomas (*n* = 15, *P* < 0.05). At >10 months of age, *hTS/Men1^–/–^* mice (*n* = 18) still showed a higher frequency of carcinomas as compared with *Men1^–/–^* mice (*n* = 32) (88.9% vs. 68.8%, *P* < 0.05), but the difference was less prominent than at the earlier time periods. In summary, we observed that TS overexpression accelerated carcinoma onset as early as 5 months of age, demonstrating an onset of carcinoma 3 months earlier in *hTS/Men1^–/–^* as compared with *Men1^–/–^* mice. These data suggest that the reduction in overall survival of *hTS/Men1^–/–^* as compared with *Men1^–/–^* control mice (Figure 1) arises due to enhanced hTS-mediated acceleration of tumor progression.

**Figure 3:**
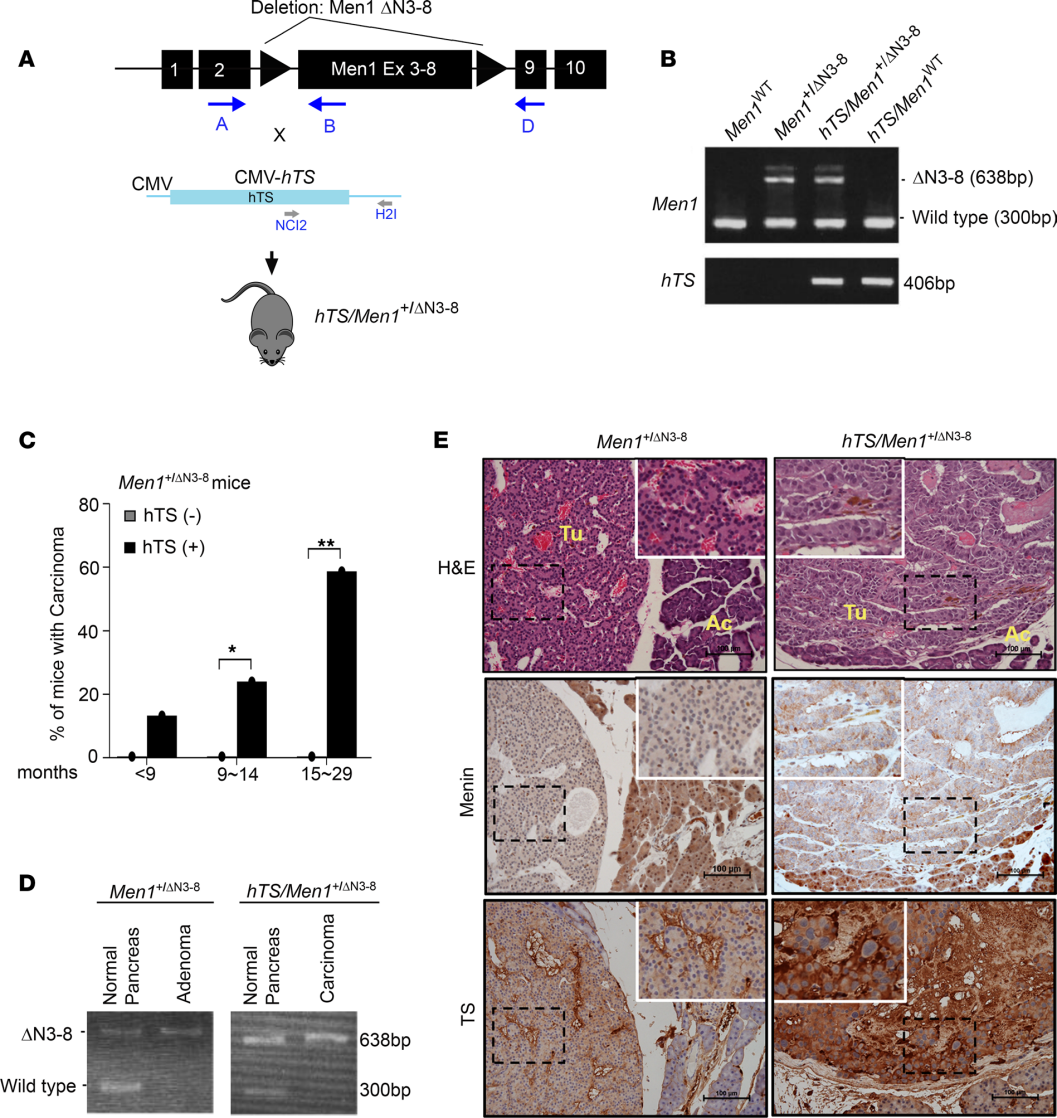
Transgenic expression of hTS increases incidence of PanNETs in mutant *Men1* mice. (**A**) Schematic representation of *hTS/Men1^+/ΔN3-8^* mouse model generation. Forward and reverse primers for the detection of *Men1* deletion and *hTS* transgene expression are shown with arrows. (**B**) Representative PCR analysis of genomic DNA from mouse tail. *Men1^+/ΔN3-8^* and *hTS/Men1^+/ΔN3-8^* mice show mutant 638 bp and WT 300 bp band. Presence of hTS in both *hTS/Men1^+/ΔN3-8^* mice and *hTS/Men1*^WT^ is shown by a 406 bp band. (**C**) Comparison of islet carcinoma incidence in *Men1^+/ΔN3-8^* and *hTS/Men1*^+/ΔN3-8^ mice. Significance of difference in the incidence of carcinoma between *hTS/Men1^+/ΔN3-8^* and *Men1^+/ΔN3-8^* mice at ages 9–14 months (*P = 0.028) and 15–29 months (**P = 0.002) was calculated by Fisher’s exact test. (**D**) PCR analysis showing loss of heterozygosity of *Men1* in pancreatic tissue isolated from *Men1^+/ΔN3-8^* and *hTS/Men1^+/ΔN3-8^* mice. (**E**) H&E and IHC staining of TS and menin in *Men1^+/ΔN3-8^* and *hTS/Men1^+/ΔN3-8^* mouse pancreatic tumor. Scale bars: 100 μm. Rectangular regions within dashed lines are enlarged (×1.8) and shown within solid lines. Ac, acinar cells; Tu, tumors.

Since heterozygous *Men1^+/–^* mice developed a spectrum of endocrine tumors similar to those observed in human patients with MEN1 syndrome (8, 23, 24), we tested whether hTS overexpression could also promote PanNET progression in heterozygote *Men1*-mutant (*Men1*^+/*Δ*N3-8^) mice. We crossed *Men1*^+/*Δ*N3-8^ with the *hTS*-transgenic mouse (8, 12) (Figure 3A) and confirmed its heterozygous *Men1*^+/*Δ*N3-8^ status as well as presence of *hTS* (Figure 3B). The entire pancreas was collected for histopathological analysis from mice that were euthanized at defined time points as well as at survival end point, and each pancreas was scored by a masked veterinary pathologist according to criteria previously described (22). The neuroendocrine origin of tumors derived from *hTS/Men1*^+/*Δ*N3-8^ and control mice was verified by confirmation of expression of synaptophysin and chromogranin A by IHC (Supplemental Figure 1). Comparison of the histological features of islet lesions between *Men1*^+/*Δ*N3-8^ and *hTS/Men1*^+/*Δ*N3-8^ mice for each age group showed that hTS overexpression had no effect on adenoma formation in *hTS/Men1*^+/*Δ*N3-8^ mice (Supplemental Table 3). However, we observed enhanced carcinoma development in *hTS/Men1*^+/*Δ*N3-8^ mice as compared with *Men1*^+/*Δ*N3-8^ controls (Supplemental Table 3). We found that *hTS* overexpression induced carcinoma development in 13.3% of *hTS/Men1*^+/*Δ*N3-8^ mice as early as 9 months of age (*P* = 0.28) with a 58.7% increase in carcinoma by <29 months (*P* = 0.002), while carcinoma was not observed in *Men1*^+/*Δ*N3-8^ mice (Figure 3C and Supplemental Table 3).

Heterozygote *Men1*-mutant (*Men1*^+/*Δ*N3-8^) mice developed pancreatic islet cell adenoma after loss of WT *Men1* allele, similar to observations in sporadic human PanNETs (6). Since *Men1*^+/*Δ*N3-8^ mice did not develop carcinoma when tested up to 21 months of age (Figure 3C), we asked whether loss of heterozygosity (LOH) occurs in *Men1*^+/*Δ*N3-8^ mice that develop adenoma as well as in *hTS/Men1*^+/*Δ*N3-8^ mice that develop carcinoma by 9 months of age. PCR and IHC analysis were performed on pancreatic tissues from *hTS/Men1*^+/*Δ*N3-8^ and *Men1*^+/*Δ*N3-8^ control mice. DNA was isolated from islet cell tumors and from surrounding normal pancreas from the same mouse. PCR analysis showed both WT and mutant *Men1* bands in adjacent matched normal pancreas, while tumors isolated from *Men1*^+/*Δ*N3-8^ and *hTS/Men1*^+/*Δ*N3-8^ mice showed only the 638 bp mutant *Men1* bands, indicating loss of the WT allele (Figure 3D). To confirm the loss of menin expression, we prepared sections from the pancreas of 21-month-old *Men1*^+/*Δ*N3-8^ and *hTS/Men1*^+/*Δ*N3-8^ mice. H&E staining confirmed adenoma in *Men1*^+/*Δ*N3-8^ and carcinoma in *hTS/Men1*^+/*Δ*N3-8^ mice (Figure 3E). IHC analysis showed menin loss in both adenoma and carcinoma from *Men1*^+/*Δ*N3-8^ and *hTS/Men1*^+/*Δ*N3-8^ mice, respectively, whereas intact menin expression was observed in normal islets (Figure 3E). In addition, weak TS expression was observed in adenoma while high TS levels were observed in carcinoma (Figure 3E). Our data show that loss of expression of WT *Men1* allele with loss of menin expression in adenomatous tumors in *Men1*^+/*Δ*N3-8^ mice did not progress to carcinoma (Figure 3C and Supplemental Table 3). In contrast, LOH in *hTS/Men1*^+/*Δ*N3-8^ mice resulted in carcinoma with high penetrance in the pancreas, expressing high levels of ectopic hTS.

### hTS overexpression alters cell growth in Men1-null murine tumors and human PanNET cells.

**Figure 4:**
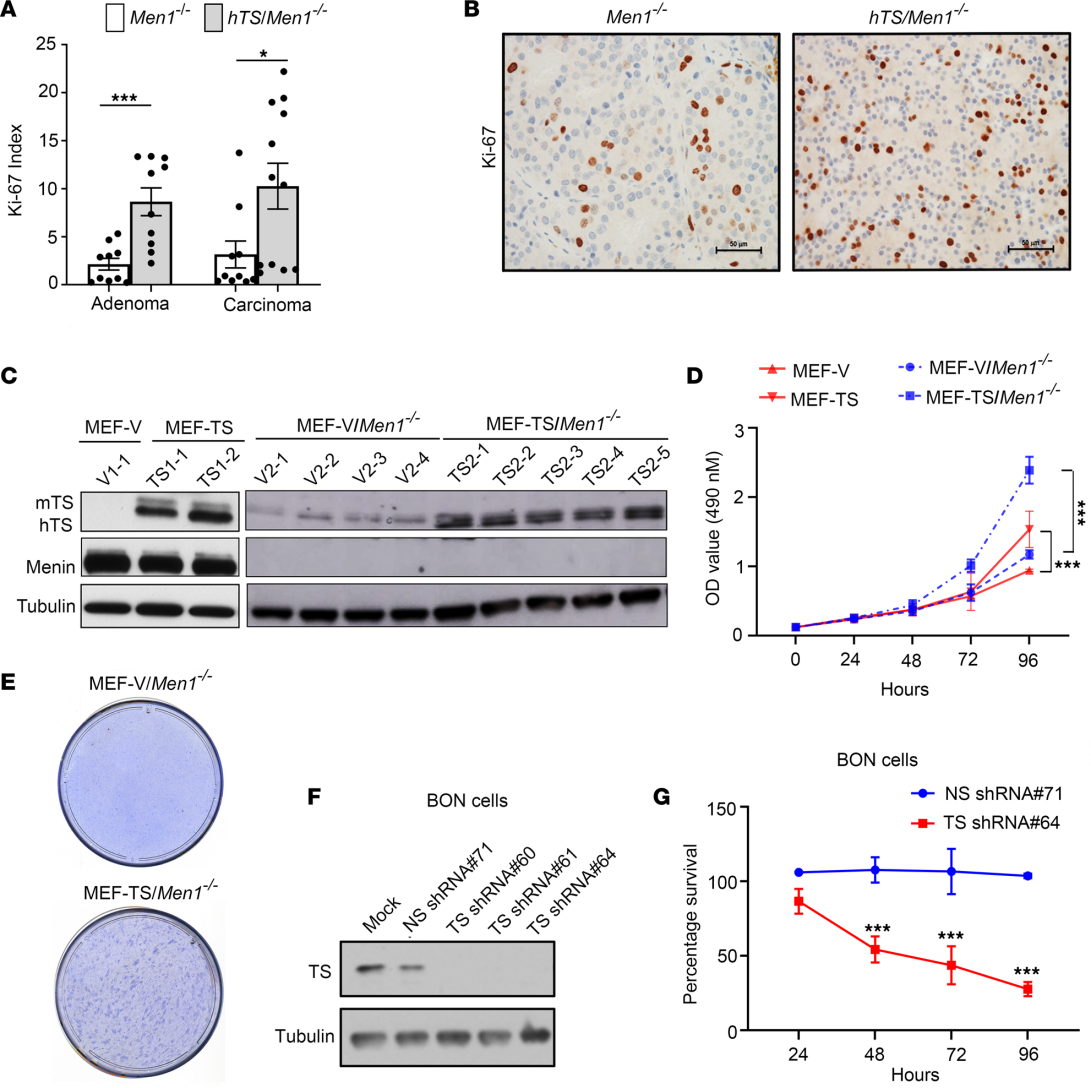
hTS overexpression alters cell growth in murine *Men1*-deleted tumor cells and in human PanNET cells. (**A**) Quantification of Ki-67 index in 10 adenoma lobules from *Men1^–/–^* mice as compared with 10 adenoma lobules from *hTS/Men1^–/–^* mice (***P = 0.0006) and 10 carcinoma lobules from *Men1^–/–^* as compared with 12 lobules from *hTS/Men1^–/–^* mice (*P = 0.024). At least 500 cells from each lobule were analyzed by ImageJ software. Significance was calculated by 2-tailed Student’s *t* test; data represent mean ± SEM. (**B**) Representative image of islet adenoma tissues of *Men1^–/–^* and *hTS/Men1^–/–^* mice immunostained with Ki-67 (scale bar: 50 μm). (**C**) TS and menin expression in cell clones established from MEF WT and MEF/*Men1^–/–^* cells transfected with hTS or vector control (V). (**D**) TS overexpression increases the growth rate of MEF-TS and MEF-TS/*Men1^–/–^* cells as compared with MEF-V and MEF-V/*Men1^–/–^* control cells, respectively, by MTS assay. Significance was calculated by 2-way ANOVA followed by Tukey’s multiple comparisons, ***P < 0.0001. (**E**) Representative picture of foci formed in MEF-TS/*Men1^–/–^* cells compared with MEF-V/*Men1^–/–^* cells after 4 weeks of growth. (**F**) TS expression in BON cells transduced with different TS shRNAs or nonspecific (NS) shRNA. In **C** and **F**, tubulin was used as loading control. (**G**) Cell viability of BON cells transduced in triplicates with TS shRNA or NS shRNA and analyzed at different time points by MTS assay. Significance was calculated by 2-way ANOVA followed by Tukey’s multiple comparisons, ***P < 0.0001. In **D** and **G**, data represent mean ± SD.

Since overexpression of hTS in the *Men1*-null mouse accelerated PanNET progression, we reasoned that elevated TS level also leads to the proliferation of PanNET cells. To evaluate the role of elevated hTS in promoting tumor cell proliferation, we analyzed Ki-67 expression in adenoma and carcinoma isolated from both *Men1^–/–^* and *hTS/Men1^–/–^* mice. Pancreatic adenoma and carcinoma tissues isolated from *Men1^–/–^* (*n* = 4) and *hTS/Men1^–/–^* mice (*n* = 5) were subjected to immunostaining with anti–Ki-67. Five adenoma lobules from the same tumor section harvested from each of the *Men1^–/–^* (*n* = 2) and *hTS/Men1^–/–^* (*n* = 2) mice with adenoma were scored and compared for Ki-67 expression. Similarly, carcinoma lobules from the same tumor section isolated from *Men1^–/–^* (*n* = 2; 10 lobules) and *hTS/Men1^–/–^* (*n* = 3; 12 lobules) were also scored and compared for Ki-67 expression. We found a 4-fold increase in Ki-67 index in adenoma isolated from *hTS/Men1^–/–^* mice compared with adenoma from *Men1^–/–^* mice (*P* = 0.0006) and a 3.2-fold increase of Ki-67 index in carcinoma isolated from *hTS/Men1^–/–^* mice when compared with carcinoma from *Men1^–/–^* mice (*P* = 0.024) (Figure 4A). Taken together, these results suggest an increased proliferation of PanNET cells due to overexpression of hTS. A representative image of islet adenoma tissues of *Men1^–/–^* and *hTS/Men1^–/–^* mice immunostained with anti–Ki-67 is presented to show the increased cell proliferation in hTS-overexpressed PanNET tissues (Figure 4B).

To test the growth-promoting efficacy of hTS in vitro, we expressed ectopic hTS in WT and *Men1*-null mouse embryo fibroblast (MEF) cells. WT MEF cells were stably transfected with hTS expression plasmid or empty vector (V) to generate MEF-TS and MEF-V cells, respectively. Six stable clones of MEF-TS (designated as TS1-1, TS1-2, TS1-3, TS1-4, TS1-5, and TS1-6) and 2 stable clones of MEF-V cells (designated as V1-1 and V1-2) were established (Figure 4C and Supplemental Figure 2). *Men1*-null MEF cells were also transfected with hTS expression plasmid or empty vector to generate MEF-TS/*Men1^–/–^* and MEF-V/*Men1^–/–^* cells, respectively. Stable clones of MEF-TS/*Men1^–/–^* (designated as TS2-1, TS2-2, TS2-3, TS2-4, and TS2-5) and MEF-V/*Men1^–/–^* cells (designated as V2-1, V2-2, V2-3, and V2-4) were developed (Figure 4C). The levels of TS in representative MEF–TS-overexpressing clones were verified through immunoblotting (Figure 4C and Supplemental Figure 2).

To determine whether overexpression of hTS alters the growth properties of MEF cells with and without *Men1* deletion, the proliferation of MEF-TS and MEF-TS/*Men1^–/–^* cells was compared with that of vector-alone control cells using MTS assay. First, we analyzed the influence of TS overexpression on the proliferation of MEF-TS cells by comparing the growth rate of MEF-TS cells versus MEF-V cells as described in Supplemental Methods. We observed a 1.5-fold increase (*P* = 0.0001) in the proliferation of MEF-TS compared with MEF-V cells at 96 hours after plating (Figure 4D). We next tested whether overexpression of hTS altered the growth of MEF-TS/*Men1^–/–^* cells by comparing the proliferation of MEF-TS/*Men1^–/–^* versus MEF-V/*Men1^–/–^* cells as described in Supplemental Methods. The results showed a proliferative advantage of MEF-TS/*Men1^–/–^* over MEF-V/*Men1^–/–^* cells (Figure 4D). The growth difference between MEF-TS/*Men1^–/–^* and MEF-V/*Men1^–/–^* became evident at 72 hours (*P* = 0.0061) and increased 2.0-fold at 96 hours after plating (*P* = 0.0001) (Figure 4D). In contrast, the growth rate of MEF-V/*Men1^–/–^* compared with MEF-V cells showed no difference even at 96 hours after plating (*P* = 0.61), indicating that *Men1* loss alone did not cause statistically significant proliferative advantage in these cells. These results demonstrate that overexpression of TS increased proliferation in both *Men1*-WT and *Men1*-null MEF cells.

To determine whether increased growth rate due to hTS overexpression in *Men1*-null MEF cells also resulted in a transformed phenotype, we performed a foci assay and compared the efficacy of MEF-V/*Men1^–/–^* and MEF-TS/*Men1^–/–^* cells to induce transformed foci in a monolayer culture as described in Supplemental Methods. We observed that MEF-TS/*Men1^–/–^* cells formed foci in a monolayer culture within 2 weeks after plating. In contrast, no foci were observed when MEF-V/*Men1^–/–^* cells were tested up to 4 weeks in culture (Figure 4E), suggesting that elevated TS levels were required for the transformed phenotype. We also performed a foci assay to compare MEF-TS with MEF-V cells and did not observe foci formation from either of these cell clones (data not shown). Taken together, these results suggest that the transformed phenotype may require the cooperation of oncogenic hTS with mutational inactivation of *Men1* in MEF cells.

To determine whether TS levels play a role in the regulation of human neuroendocrine tumor cell growth, TS expression was inhibited in BON cells by transduction of lentiviral vectors carrying TS shRNA (designated TS shRNA#60, #61, and #64) and nonspecific shRNA control (NS shRNA#71). Immunoblot analysis showed that transduction of TS shRNA inhibited TS levels as compared with NS shRNA control (Figure 4F). To test whether reduction in TS level affected viability of BON cells, we performed an MTS assay following transduction of TS shRNA and NS shRNA control. We observed statistically significant reduction in the proliferation of BON cells 48 hours after TS shRNA transduction as compared with NS shRNA (*P* < 0.0001). The reduction in proliferation was further increased at 72 hours (*P* < 0.0001) and 96 hours following transduction (*P* < 0.0001) (Figure 4G). In summary, TS levels regulate proliferation of MEF and human PanNET cells.

To address whether TS overexpression arises as a coordinated cellular program regulating nucleotide metabolism that may promote cell proliferation, we analyzed the expression of genes involved in nucleotide metabolism and their relationship with TS gene expression using data available through the NCBI Gene Expression Omnibus (GEO) database as described in Supplemental Methods. We selected 21 genes that regulate purine, pyrimidine, and folate synthesis and observed that expression of 7 of 21 genes positively correlated with TS levels in human PanNETs (Supplemental Figure 3). These included genes regulating the folate cycle (*DHFR* and *MTHFD1*), purine synthesis (*PRPS2*), and pyrimidine synthesis (*TK1* and *DTYMK*) and genes regulating both purine and pyrimidine synthesis (*RRM1* and *RRM2*). More work is required to determine whether there is a coordinated modulation within a subset of genes involved in nucleotide synthesis and TS overexpression.

We also asked whether TS overexpression induces global metabolic changes in distinct metabolic pathways, specifically those involved in nucleotide synthesis. We analyzed the metabolomic profiles of our control WT mouse embryonic fibroblasts (MEF-V) and *Men1^–/–^* (MEF-V/*Men1^–/–^*) with and without TS overexpression (described in Supplemental Methods), and we observed that MEFs overexpressing TS (MEF-TS and MEF-TS/*Men1^–/–^*) compared with MEF-V and MEF-V/*Men1^–/–^* showed upregulation of metabolic pathways involved in nucleotide biosynthesis, amino acid metabolism, sugar and lipid biosynthesis, and nitrogen metabolism. As expected, the pyrimidine pathway was the most upregulated metabolic pathway with TS overexpression. Purine metabolism was also found to be upregulated in TS-overexpressing cells (Supplemental Figure 4), suggesting that TS overexpression induces global metabolic changes, particularly in pathways involved in nucleotide biosynthesis. A detailed mechanistic study linking TS overexpression and nucleotide synthesis as well as global metabolomics using animal models and patient samples will be conducted in the future.

### Overexpression of hTS regulates expression of CDK inhibitors and induces G_2_/M phase accumulation in Men1-null cells.

**Figure 5:**
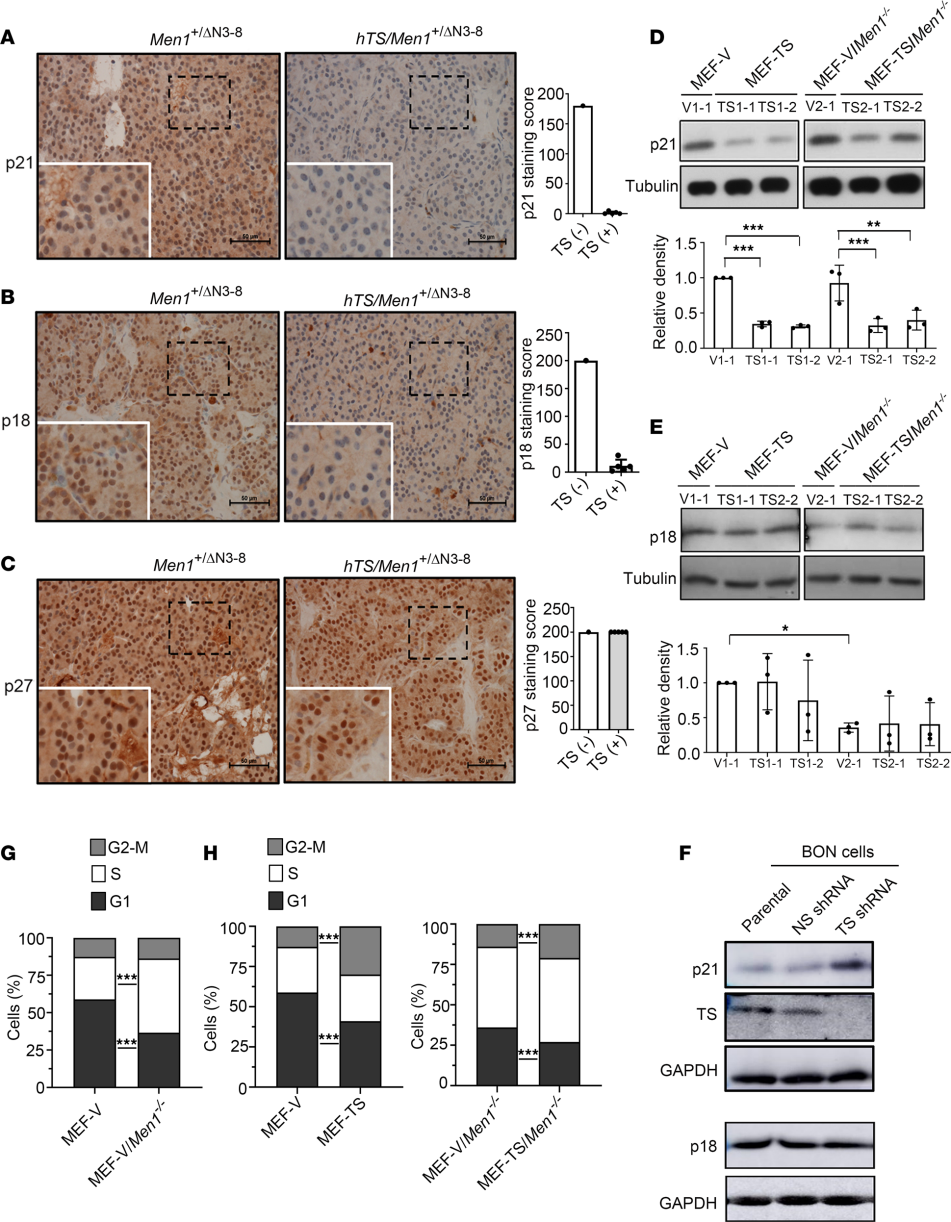
Effect of hTS on expression of CDK inhibitors and cell cycle regulation. (**A**–**C**) Representative IHC analysis of p21^Cip1^, p18^INK4c^, and p27^Kip1^ in PanNET tissue sections of *hTS/Men1^+/ΔN3-8^* (*n* = 5) and *Men1^+/ΔN3-8^* (*n* = 1) mice. Scale bars: 50 μm. Rectangular regions within dashed lines are enlarged (×1.8) and shown within solid lines. Quantification of immunostaining is shown by staining score for p21^Cip1^, p18^INK4c^, and p27^Kip1^. (**D** and **E**) Representative p21^Cip1^ and p18^INK4c^ immunoblot and relative densitometry quantification in MEF-TS, MEF-TS/*Men1^–/–^*, and vector controls (MEF-V, MEF-V/*Men1^–/–^*) (*n* = 3 independent experiments per clone). Significance was calculated by 2-way ANOVA with multiple comparisons using the 2-stage linear step-up procedure of Benjamini, Krieger, and Yekutieli, *P < 0.029, **P = 0.0004, ***P < 0.0001. In **A**–**E**, data represent mean ± SD. (**F**) Immunoblot showing expression of TS, p21^Cip1^, and p18^INK4c^ in parental BON cells and in BON cells transduced with NS shRNA#128 and TS shRNA#133. Tubulin (**D** and **E**) or GAPDH (**F**) was used as loading control. (**G**) Cell cycle distribution pattern of MEF-V versus MEF-V/*Men1^–/–^* cells using flow cytometry. (**H**) Cell cycle distribution pattern of MEF-V, MEF-TS, MEF-V/*Men1^–/–^*, and MEF-TS/*Men1^–/–^* cells using flow cytometry. In **G** and **H**, data represent the mean values obtained from 2–4 independent flow cytometric assays using 2 distinct clones for each cell type, and significance was calculated by 2-tailed Student’s *t* test, ***P < 0.0001.

The CDK inhibitors p21^Cip1^, p18^INK4c^, and p27^Kip1^ are tumor suppressors that regulate cell cycle kinetics (25–28). *Men1* loss is known to downregulate p18^INK4c^ and p27^Kip1^, which promotes cell cycle progression and tumor development in islet cell tissues (29). TS is also reported to regulate the cell cycle through modulation of p21^Cip1^ in MCF-7 cells through an undefined mechanism (30). To gain insight into the mechanism underlying the ability of hTS to promote tumorigenesis in *Men1*-null mice, we performed IHC analysis to examine p21^Cip1^, p18^INK4c^, and p27^Kip1^ expression in PanNET tissues isolated from *hTS/Men1*^+/*Δ*N3-8^ mice (*n* = 5; 2 adenoma and 3 carcinoma) compared with control PanNET tissues isolated from *Men1*^+/*Δ*N3-8^ mice (*n* = 1; adenoma). We observed loss of p21^Cip1^ expression and decrease of p18^INK4c^ expression in PanNET *hTS/Men1*^+/*Δ*N3-8^ tumor tissues (p21^Cip1^ absent in 5/5 tissues, mean staining score = 5 as described in Methods; p18^INK4c^ reduced or absent in 3/5 tissues, mean staining score = 11.4) compared with age-matched tissues from *Men1*^+/*Δ*N3-8^ mice (p21^Cip1^ staining score = 180 and p18^INK4c^ staining score = 200) (Figure 5, A and B), suggesting a role for hTS overexpression in downregulating p21^Cip1^ and to a lesser extent downregulating p18^INK4c^ levels. However, no change in p27^Kip1^ expression was observed in PanNET tissue from *hTS/Men1*^+/*Δ*N3-8^ mice when compared with control *Men1*^+/*Δ*N3-8^ tumor tissue (mean staining score = 200 for both *hTS/Men1*^+/*Δ*N3-8^ and *Men1*^+/*Δ*N3-8^ mouse tissue) (Figure 5C). We also observed loss of p21^Cip1^ and p18^INK4c^ expression in pancreatic islet tumors from both *Men1^–/–^* (*n* = 4; 2 adenoma and 2 carcinoma) and *hTS/Men1^–/–^* mice (*n* = 4; 2 adenoma and 2 carcinoma), while p27^Kip1^ expression was not affected (Supplemental Figure 5). Therefore, homozygous *Men1* loss is associated with reduced expression of p21^Cip1^ and p18^INK4c^, with no additional effect shown from elevated TS levels. Since pancreatic tissues from *Men1^–/–^* but not *Men1*^+/*Δ*N3-8^ mice showed absence of p21^Cip1^ and p18^INK4c^ expression, we asked whether the WT *Men1* allele was retained in the *Men1*^+/*Δ*N3-8^ tumor samples. We observed LOH in both *Men1*^+/*Δ*N3-8^ and *hTS/Men1*^+/*Δ*N3-8^ mouse pancreas; however, only *hTS/Men1*^+/*Δ*N3-8^ mice showed loss of p21^Cip1^ and partial loss of p18^INK4c^ expression. Taken together, these results suggest that overexpression of hTS downregulates CDK inhibitors p21^Cip1^ and p18^INK4c^ in islet tumors of *hTS/Men*^+/*Δ*N3-8^ mice, whereas homozygous deletion of *Men1* alone is sufficient for the loss of p21^Cip1^ and p18^INK4c^ expression in *Men1^–/–^* mice independent of hTS levels.

To determine whether TS overexpression can modulate CDK inhibitors in *Men1*-null and *Men1*-WT MEF cells, we performed immunoblot analysis to determine p21^Cip1^ and p18^INK4c^ expression in MEF-TS and MEF-TS/*Men1^–/–^* compared with matched empty vector control MEF cells (MEF-V and MEF-V/*Men1^–/–^*). We observed a decrease in p21^Cip1^ expression in both MEF-TS and MEF-TS/*Men1^–/–^* as compared with MEF-V and MEF-V/*Men1^–/–^* control cells, respectively (TS1-1 vs. V1-1, *P* < 0.0001; TS1-2 vs. V1-1, *P* < 0.0001; TS2-1 vs. V2-1, *P* < 0.0001; and TS2-2 vs. V2-1, *P* = 0.0004) (Figure 5D and additional clones shown in Supplemental Figure 6A). However, this decrease in p21^Cip1^ expression was not observed in MEF-V/*Men1^–/–^* cells as compared with MEF-V cells (Figure 5D and Supplemental Figure 6A). These results suggest that the ability of TS overexpression to downregulate p21^Cip1^ expression in vitro is independent of *Men1* status. In contrast, overexpression of TS did not reduce p18^INK4c^ levels in MEF-TS and MEF-TS/*Men1^–/–^* cells when compared with empty vector controls (Figure 5E). A reduction of p18^INK4c^ expression was observed in MEF-V/*Men1^–/–^* cells when compared with MEF-V cells (0.4-fold, *P* = 0.029) (Figure 5E and Supplemental Figure 6B). These results suggest an association between *Men1* deletion and a lowering p18^INK4c^ expression regardless of hTS level. In summary, TS overexpression was associated with reduced p21^Cip1^ expression, while deletion of *Men1* was found to downregulate p18^INK4c^ expression in MEF cells. The role of TS modulating p21 and p18^INK4c^ expression was further studied in BON cells, a metastatic human PanNET cell line. BON cells were stably transduced with lentiviral TS shRNA (designated #133) or with nonspecific (NS) shRNA (designated #128). Immunoblot analysis showed reduction of TS levels using TS shRNA as compared with transduction with NS control shRNA (Figure 5F). We found that p21^Cip1^ was elevated in cells transduced with TS shRNA in comparison with control cells while the levels of p18^INK4c^ remained unaltered (Figure 5F). Taken together, these results show that TS regulates p21^Cip1^ levels in MEF and in human pancreatic neuroendocrine cells.

Since overexpression of TS or deletion of *Men1* can downregulate expression of CDK inhibitors, we tested the effect of ectopic TS on regulation of cell cycle kinetics. To study the influence of TS overexpression in the in vitro cell cycle distribution pattern of MEF-V and MEF-V/*Men1^–/–^* cells and their TS-overexpressing counterparts, we analyzed DNA content by flow cytometry as described in Supplemental Methods. These results show that high TS levels increase G_2_/M entry in MEF-TS and MEF-TS/*Men1^–/–^* cells compared with MEF-V and MEF-V/*Men1^–/–^* controls (Figure 5, G and H). We also evaluated the influence of *Men1* deletion on cell cycle pattern by comparing the cell cycle distribution between MEF-V and MEF-V/*Men1^–/–^* cells. We observed that 58.8% of MEF-V cells were distributed in G_1_ phase and 28.4% in S phase, while MEF-V/*Men1^–/–^* displayed a decreased percentage of cells in G_1_ phase (36.5%) (*P* < 0.0001) and a higher percentage in S phase (49.5%) (*P* < 0.0001) (Figure 5G). This suggests enhanced G_1_ to S phase entry with *Men1* deletion, similar to a previous report suggesting a role for *Men1* function in regulating G_1_ to S phase transition (31). We next tested the cell cycle effect in MEF-V and MEF-V/*Men1^–/–^* cells due to hTS overexpression. We observed a significant increase in G_2_/M phase of MEF-TS cells (12.6% vs. 30.5%, *P* < 0.0001), along with a concomitant decrease in the percentage of cells in G_1_ phase when compared with MEF-V cells (58.8% vs. 42.6%, *P* < 0.0001). These data suggest a G_2_/M phase arrest in hTS-overexpressing cells (Figure 5H). Similarly, overexpression of hTS in *Men1*-deleted cells also increased the G_2_/M phase progression (20.9% vs. 13.8%, *P* < 0.0001) with a decreased G_1_ phase observed in MEF-TS/*Men1^–/–^* cells when compared with MEF-V/*Men1^–/–^* cells (36.5% vs. 26.9%, *P* < 0.0001). hTS overexpression did not alter the S phase duration of MEF-TS/*Men1^–/–^*, which was already elevated as a result of *Men1* deletion (Figure 5H). In summary, hTS overexpression induces a G_2_/M phase arrest in MEF cells in the presence or absence of *Men1* deletion. The overexpression of hTS did not further alter the increased S phase in MEF cells due to *Men1* deletion.

### Overexpression of hTS induces DNA damage and chromosomal aberrations in a Men1-deleted genetic background.

**Figure 6:**
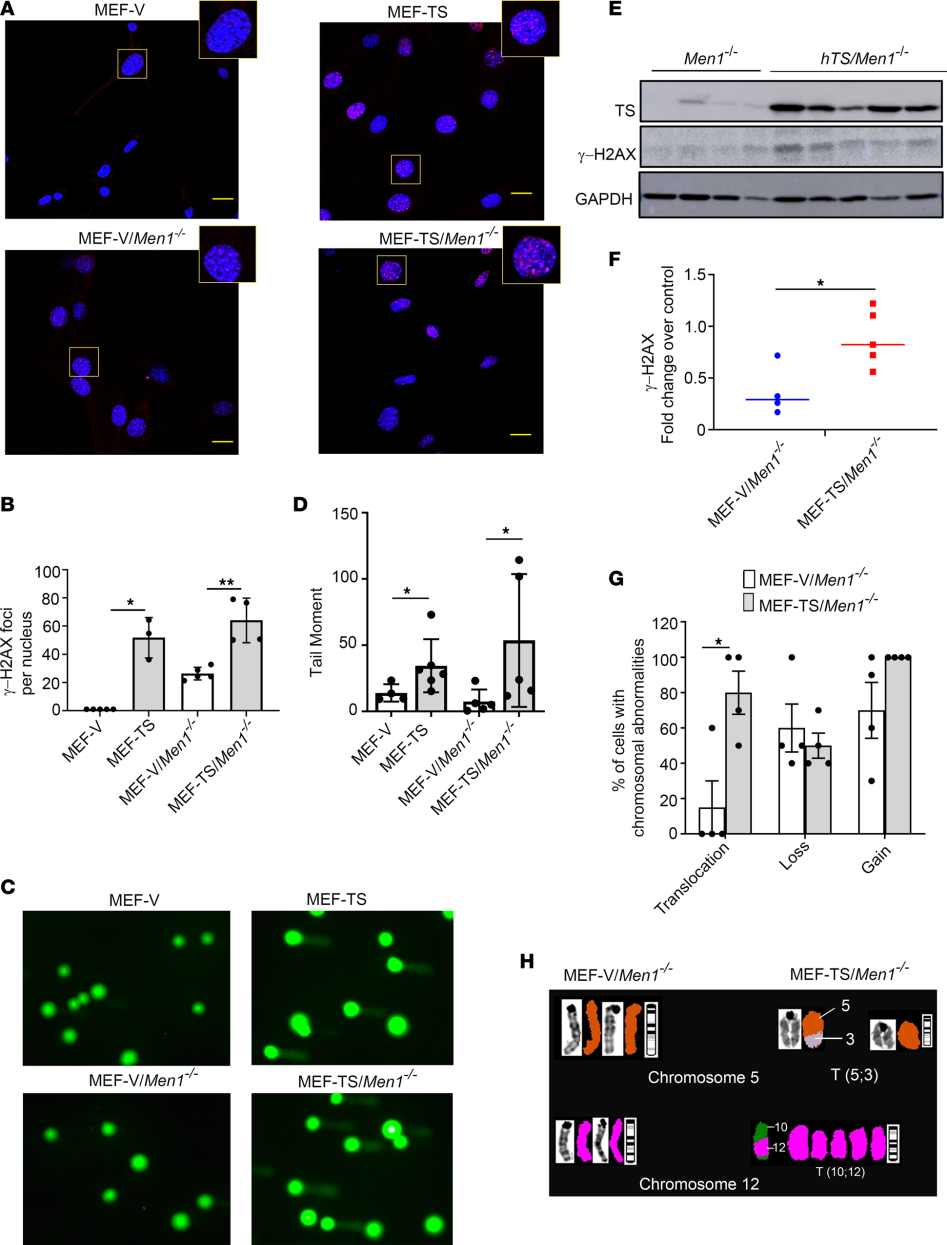
DNA damage and chromosomal instability in MEF-TS/*Men1^–/–^* cells. (**A**) Immunofluorescence staining of γ-H2AX in MEF-TS, MEF-TS/*Men1^–/–^*, MEF-V, and MEF-V/*Men1^–/–^* control cells (scale bars: 10 μm). Rectangular region is shown enlarged ×2. (**B**) Quantification of γ-H2AX foci obtained with ImageJ software (NIH) from 3 independent experiments. Significance was calculated by 2-tailed Student’s *t* test, *P < 0.0035, **P < 0.0013. (**C**) Representative image of a comet assay using MEF-TS and MEF-TS/*Men1^–/–^* cells and their corresponding controls. (**D**) Quantification of the tail moment with each data point representing the average tail moment of at least 10 cells from a single independent experiment. For each group, at least 2 clones were analyzed, and 2–3 independent experiments were conducted for each clone. *P < 0.05 calculated by Mann-Whitney *U* test. (**E** and **F**) Immunoblot analysis of γ-H2AX expression in tumor developed from *Men1^–/–^* (*n* = 4) and *hTS/Men1^–/–^* mice (*n* = 5). Significance was calculated by 2-tailed Student’s *t* test, *P < 0.05. In **B**, **D**, and **F**, data represent mean ± SD. (**G**) Chromosomal abnormalities in MEF-TS/*Men1^–/–^* and MEF-V/*Men1^–/–^* cells detected by spectral karyotyping analysis. Ten cells in metaphase from 4 clones of MEF-TS/*Men1^–/–^* and MEF-V/*Men1^–/–^* cells were analyzed to identify the percentage of cells with chromosomal translocations, gains, and losses. *P < 0.05 calculated by 2-tailed Student’s *t* test; data represent mean ± SEM. (**H**) Representative spectral karyotype image of MEF-V/*Men1^–/–^* and MEF-TS/*Men1^–/–^* cells. Chromosomal translocations T(10;12) and T(5;3) detected only in hTS-transfected cells are shown. G-band staining for chromosome 5 of V2-1 and TS2-2 clones and for chromosome 12 of V2-1 and TS2-1 clones is shown.

Since we observed that TS overexpression induces a G_2_/M phase arrest, which is associated with DNA damage (32), we tested whether TS overexpression could enhance DNA damage in MEF-TS and MEF-TS/*Men1^–/–^* cells. To study the role of TS overexpression in DNA damage, we scored the presence of γ-H2AX foci, a molecular marker for DNA damage, in MEF-TS and MEF-TS/*Men1^–/–^* cells and compared the results with those in matched control cells. This experiment was repeated 3 times, and up to 3 immunofluorescent fields from each experiment were selected for counting of γ-H2AX foci using ImageJ software (NIH). We observed a negligible number of γ-H2AX foci in control MEF-V cells (1 focus per cell). In contrast, a statistically significant increase of γ-H2AX foci was found in MEF-TS cells (52 foci per cell; *P* = 0.0035), indicating a role for TS overexpression in the induction of DNA damage in MEF cells (Figure 6, A and B). MEF-V/*Men1^–/–^* cells also exhibited a statistically significant increase in the number of γ-H2AX foci (26 foci per cell), which was further increased in MEF-TS/*Men1^–/–^* cells (64 foci per cell; *P* = 0.0013) (Figure 6, A and B). These results indicate that hTS alone is sufficient to induce DNA damage in MEF cells and that *Men1* deletion is associated with increased DNA damage that was further enhanced by high levels of TS.

In addition, we asked whether TS overexpression could cause DNA strand breakage as measured by comet assay. DNA breaks appear as a diffuse tail, which can be quantified as tail moment (product of tail length and intensity). We performed an alkaline comet assay comparing MEF-TS and MEF-TS/*Men1^–/–^* cells with MEF-V and MEF-*Men1^–/–^* controls, respectively (see Supplemental Methods). We found that the mean tail moment of MEF-TS cells (34.4) was higher than that of MEF-V cells (13.9) (*P* = 0.019) (Figure 6, C and D). The mean tail moment of MEF-TS/*Men1^–/–^* (53.5) was also found to be higher than that of MEF-V/*Men1^–/–^* (7.3) (*P* = 0.032) (Figure 6, C and D). Through 2 independent techniques, our results demonstrate that overexpression of TS increases double-strand DNA damage in both WT and *Men1*-null cells.

DNA double-strand break lesions result in severe consequences for cell survival and the maintenance of genomic stability (33). Therefore, we tested whether overexpression of TS induced DNA damage in PanNETs by measuring expression of γ-H2AX in carcinoma developed in hTS/*Men1^–/–^* and *Men1^–/–^* mice. Using immunoblot analysis, we found that the levels of γ-H2AX were higher in tumor samples derived from *hTS/Men1^–/–^* compared with *Men1^–/–^* mice (Figure 6E). The band intensity of *hTS/Men1^–/–^* and *Men1^–/–^* mice and GAPDH was quantified by ImageJ software, and the relative expression of γ-H2AX was determined (*P* < 0.05) (Figure 6F). Our results show that TS overexpression results in DNA damage in PanNET samples in genetically engineered mouse models.

Since γ-H2AX expression and the comet assay are markers for double-stranded DNA damage that could lead to chromosomal abnormalities, genomic instability, and intracellular nucleotide imbalance due to high levels of TS (34), we asked whether TS overexpression induces chromosomal aberrations in MEF-TS/*Men1^–/–^* and MEF-*Men1^–/–^* control cells. To study whether TS-induced DNA damage leads to chromosomal translocations as well as chromosomal gains and losses in *Men1*-deleted cells, we compared the chromosomal abnormalities in MEF-TS/*Men1^–/–^* versus MEF-*Men1^–/–^* cells using spectral karyotyping analysis. Ten metaphase cells from each of 4 distinct clones of MEF-TS/*Men1^–/–^* (TS2-1, TS2-2, TS2-3, and TS2-4) and MEF-V/*Men1^–/–^* cells (V2-1, V2-2, V2-3, and V2-4) were evaluated for chromosomal abnormalities. A T(6;19) rearrangement was present in all the cell clones and was excluded from our further analyses. We observed a higher percentage of MEF-TS/*Men1^–/–^* cells with acquired chromosomal translocations compared with MEF-V/*Men1^–/–^* (80% vs. 15%; *P* < 0.05), which suggests that elevated TS may promote translocation and chromosomal instability (Figure 6G and Supplemental Tables 4 and 5). There was no statistically significant difference between the percentages of MEF-V/*Men1^–/–^* and MEF-TS/*Men1^–/–^* cells exhibiting chromosomal gains or losses (Figure 6G). In addition, we detected a T(10;12) rearrangement exclusively in MEF-TS/*Men1^–/–^* (30% of TS2-1) (Figure 6H and Supplemental Table 4). Similarly, the occurrence of chromosomal translocation T(5;3) was detected in 3 different clones of MEF-TS/*Men1^–/–^* cells (70% of TS2-2 cells, 70% of TS2-3 cells, and 50% of TS2-4 cells) and only in a single clone of control MEF-V/*Men1^–/–^* cells (10% of V2-2) (Figure 6H and Supplemental Tables 4 and 5). Taken together, these results suggest a role for TS overexpression in inducing DNA damage and chromosomal translocations in a *Men1*-deleted genetic background.

### Overexpression of hTS induces somatic mutations in a Men1-deleted mouse model.

**Figure 7:**
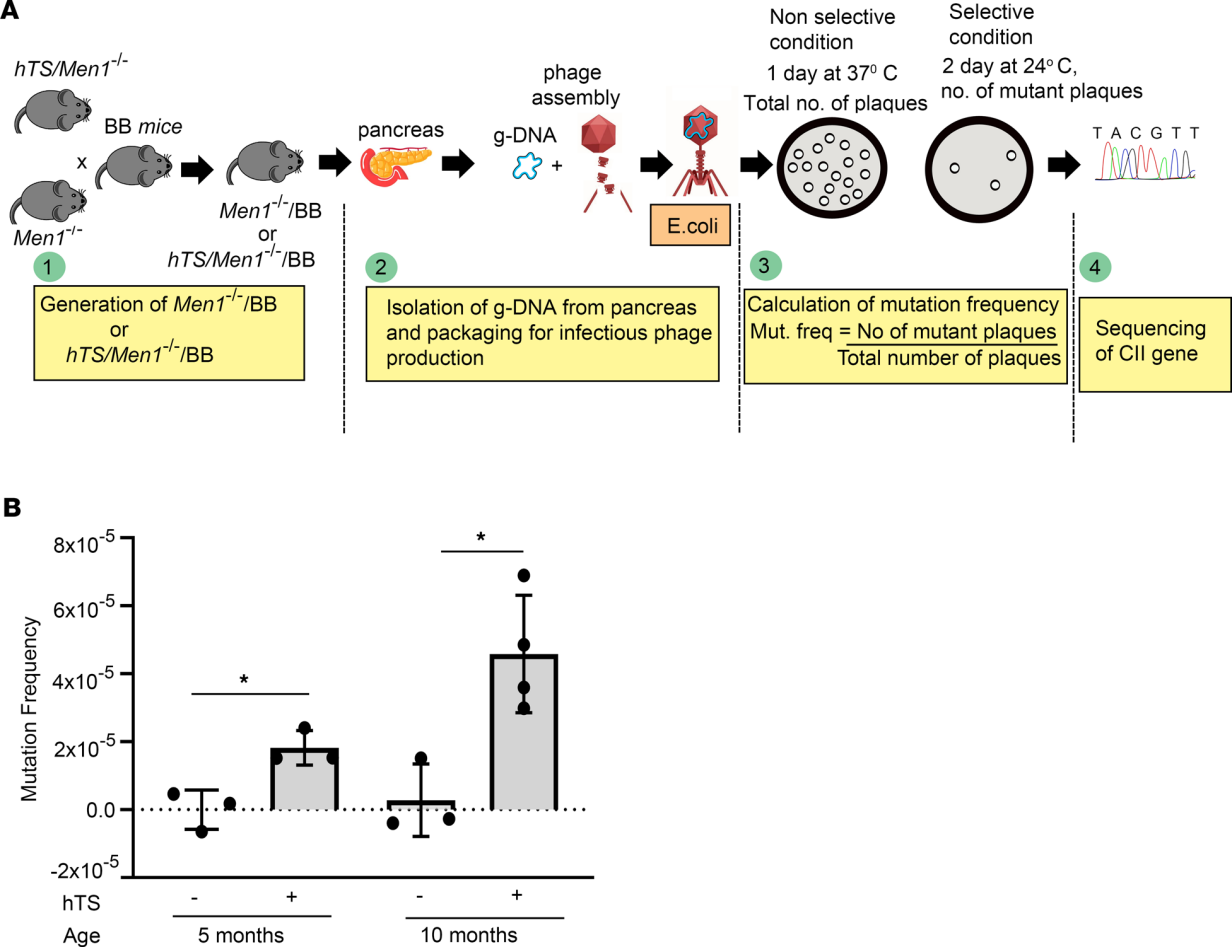
Overexpression of hTS induces somatic mutations in *Men1^–/–^*/BB mice. (**A**) Experimental scheme for the detection of somatic mutations in *hTS/Men1^–/–^*/BB and *Men1^–/–^*/BB mice by sequencing of CII gene. (**B**) Frequency of mutations in pancreas at 5 and 10 months of age. *hTS/Men1^–/–^*/BB (hTS^+^) mice (*n* = 3 mice per age group) compared with age-matched control *Men1^–/–^*/BB (hTS^–^) mice (*n* = 3 for 5-month-old and *n* = 4 for 10-month-old group). *P < 0.05 calculated by 2-tailed Student’s *t* test; data represent mean ± SD.

Since we observed an increase in markers indicative of DNA damage in MEF cells and *Men1*-null genetically engineered mouse model tumors following hTS overexpression, we tested whether hTS overexpression could induce somatic mutations in *Men1*-deleted mouse tissues. We used Big Blue model mice (BB mice, Agilent Technologies), which have multiple copies of the λ bacteriophage shuttle vector Big Blue Lambda LIZ (LacI/Z) with CII gene in its genome as the reporter for mutagenesis. The mutation in the CII gene affects the efficacy of λ phages to maintain a lysogenic phase in the bacterial host cells, which results in plaque formation at selective conditions (24°C). The BB mice were backcrossed with *Men1^–/–^* mice and *hTS/Men1^–/–^* mice to generate *Men1^–/–^*/BB and *hTS/Men1^–/–^*/BB mice, respectively. To determine the TS-induced mutations in vivo, the frequency of CII mutations in λ phages recovered from the pancreas of *hTS/Men1^–/–^*/BB mice (*n* = 7; 3 five-month-old mice and 4 ten-month-old mice) and *Men1^–/–^*/BB mice (*n* = 6; 3 five-month-old mice and 3 ten-month-old mice) was calculated based on the plaque-forming frequency of the recovered phages at 24°C (Figure 7A). We observed increased mutation frequency in phages recovered from both 5- and 10-month-old *hTS/Men1^–/–^*/BB animals, compared with phages recovered from age-matched *Men1^–/–^*/BB mice (1.81 × 10^–5^ vs. 0, *P* = 0.014, for 5-month-old mice and 4.8 × 10^–5^ vs. 2.8 × 10^–6^, *P* = 0.013, for 10-month-old mice) (Figure 7B). Subsequent sequencing of the mutated CII genes isolated from the plaques identified the specific nucleotide changes associated with TS overexpression. The most frequently occurring TS-induced variants in both 5- and 10-month-old mice were GC-to-AT transitions (66 of 362 mutations in 5-month-old mice and 187 of 831 mutations in 10-month-old mice), followed by GC-to-CG and AT-to-CG transversions (Supplemental Table 6). The mutational spectra of CII genes recovered from *hTS/Men1^–/–^*/BB and *Men1^–/–^*/BB mice were generated by R software (35) using the data obtained from sequencing of CII genes (Supplemental Figure 7). The results showed that these mutations are not enriched at specific sequences of CII genes but found spread throughout the loci, indicating that elevated TS did not induce mutational hot spots in CII genes. These results suggest that hTS overexpression can promote mutagenesis by accelerating somatic mutations in *Men1*-deleted PanNET tissues.

### TS expression level in human PanNETs is correlated with patient outcome and survival.

**Figure 8:**
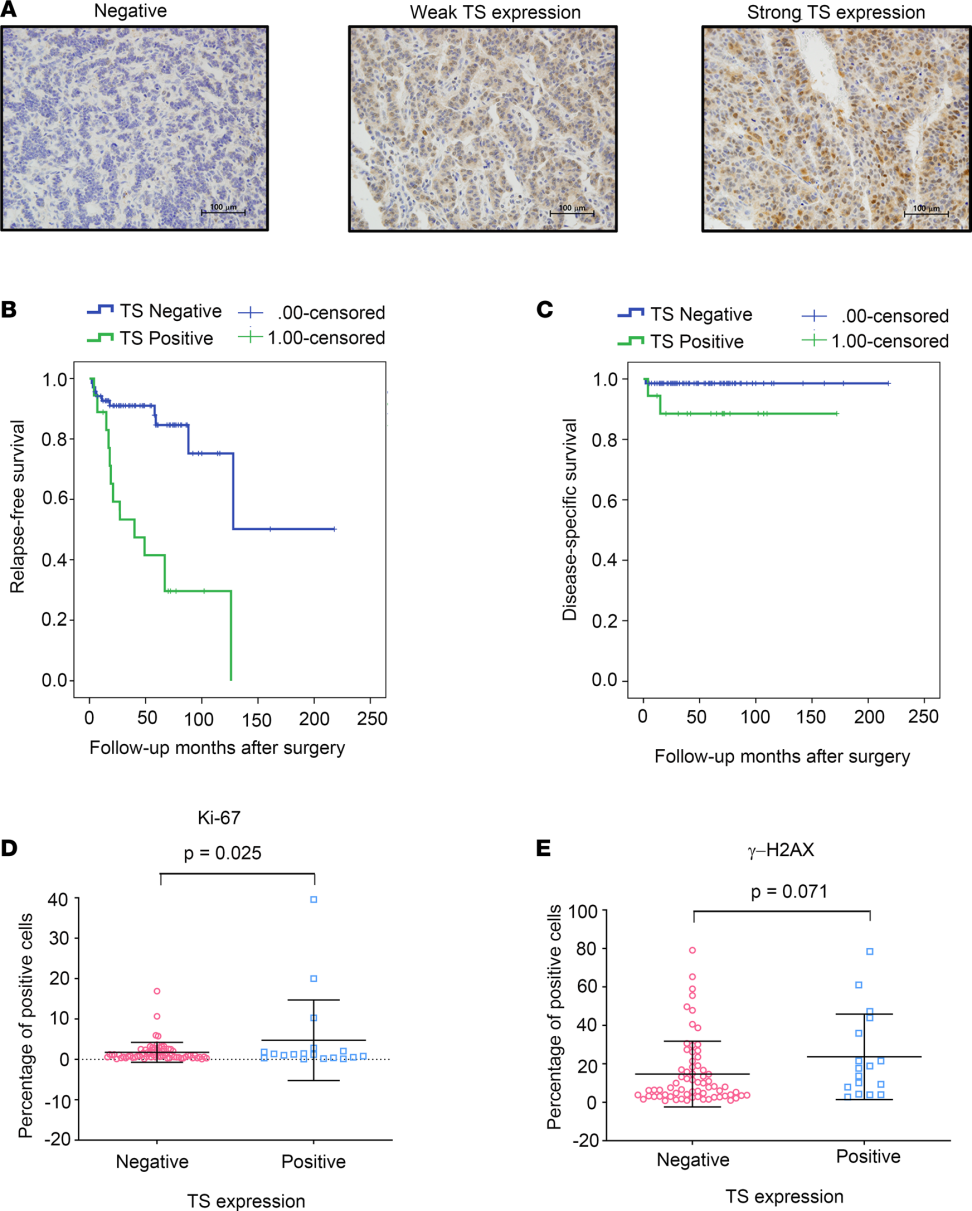
TS expression level in human PanNETs is correlated with patient outcome and survival. (**A**) IHC staining of TS in human PanNET tissues (*n* = 88). Representative images from 3 different patients showing negative, weak, and strong expression of TS. Scale bars: 100 μm. (**B** and **C**) Univariate survival analysis for patients with positive (*n* = 18) and negative (*n* = 70) TS expression. Patients with positive TS biopsies had worse relapse-free survival (*P* < 0.001) and disease-specific survival (*P* = 0.049) than those with negative TS expression. (**D** and **E**) Percentage of cells expressing Ki-67 and γ-H2AX in human PanNET samples with and without detectable TS expression as measured by IHC. Significance was calculated by 2-tailed Student’s *t* test (*P* = 0.025 for Ki-67 and *P* = 0.071 for γ-H2AX); data represent mean ± SD.

To investigate the expression level of TS in human PanNETs, we examined TS protein levels in a large series of patients with PanNETs that are also clinically annotated for survival. A bank of 88 human pancreatic endocrine tumors was studied and IHC was performed as described in Supplemental Methods. A representative picture of negative, low to moderate, and strong TS expression as determined by IHC analysis is shown in Figure 8A. TS protein was absent in normal pancreas, whereas 20.45% of clinical specimens (18/88) showed increased TS protein expression. Among the clinical specimens, 17.0% of PanNETs (15/88) exhibited low to moderate TS expression, and 3.4% of clinical specimens (3/88) strongly expressed TS protein. We did not detect TS expression in normal human pancreas tissue including acinar, ductal, and islet tissues. Using immunoblot analysis, we were also able to demonstrate enhanced TS expression in most archived human tumor samples (15) compared with immediately adjacent normal tissues (Supplemental Figure 8).

Univariate survival analysis was performed to determine whether a correlation exists between TS protein expression and patient outcome. We found that patients with low to moderate and strong expression of TS had worse outcome as determined by relapse-free survival (*P* < 0.001) and disease-specific survival (*P* = 0.049) in comparison with those with negative expression of TS (Figure 8, B and C). Using multivariate Cox regressional hazard analysis, we found that TS expression status predicted patient outcome in relapse-free survival (*P* = 0.001) independently of WHO classification but not of overall survival (*P* = 0.240). TS expression in human pancreatic endocrine tumors was significantly associated with large tumor size (*P* = 0.046) and with PanNETs of higher histological grade (WHO grade 3) (*P* = 0.026) (Supplemental Table 7).

In addition, we performed IHC analysis on the same human tumor tissues for γ-H2AX and Ki-67 expression and compared the γ-H2AX and Ki-67 expression status (measured by IHC) from human tumor samples with negative and positive TS expression. We found a modest but statistically significant increase in Ki-67 expression in TS-positive as compared with TS-negative tumor samples (*P* = 0.025; Figure 8D). γ-H2AX expression in TS-overexpressing human tumors was marginally significant as compared with that in TS-negative tumors (*P* = 0.071; Figure 8E). Taken together, our data demonstrate that overexpression of TS in human PanNETs results in worse clinical outcome in comparison with patients with negative expression of TS. In addition, our results suggest that TS can influence survival, tumor grade, and proliferation of human PanNETs.

## Discussion

Mouse models with conditional deletion of *Men1* recapitulate the clinical features of PanNETs, including a long latency (9), suggesting that additional oncogenic events are required for tumor promotion. For example, *MEN1* mutations are among the most common somatic mutations observed in human PanNETs (3, 36); however, the cooperating events in tumorigenesis are not yet defined. We have now shown that overexpression of hTS accelerates PanNET progression in *Men1*-mutated background using 3 different newly generated *hTS/Men1^+/–^*, *hTS/Men1^–/–^*, and *hTS/Men1*^+/*Δ*N3-8^ mouse models. Using these mouse models, we showed that overexpression of hTS in inactivated *Men1* islet cells shortened the latency for tumor development and reduced survival of both *hTS/Men1^+/–^* and *hTS/Men1^–/–^* mice. Scheduled necropsy of pancreatic tissues isolated from *hTS/Men1^–/–^* and *hTS/Men1*^+/*Δ*N3-8^ mice showed that hTS overexpression accelerates hyperplasia, adenoma, and islet cell carcinoma in comparison with control *Men1^–/–^* and *Men1*^+/*Δ*N3-8^ mice. Our observation that high TS levels shortened survival of *hTS/Men1^–/–^* and *hTS/Men1^+/–^* mice extends our previous work showing that TS expression in patients with TS-positive gastroenteropancreatic neuroendocrine tumors had worse outcome in comparison with patients with negligible TS expression as determined by univariate and multivariate survival analysis (15). In the current study, we also analyzed 88 patients with PanNET tumor biopsies that were annotated for TS protein levels. We demonstrated that patients with TS-positive biopsies had worse clinical outcome, and also that TS expression correlated with Ki-67 levels, supporting the hypothesis that TS may play a direct role in promoting tumorigenesis of PanNETs.

We previously demonstrated that normal, WT mice with *hTS* transgene expression developed islet cell adenoma with low penetrance and a long latency of 9–24 months (12). Data presented in this report now show that islet cell hyperplasia progresses with high penetrance through adenoma to carcinoma stage within 6 months in *hTS/Men1^–/–^* mice, whereas progression to carcinoma was not observed in age-matched *Men1^–/–^* mice. While elevated hTS alone is a weak oncogenic factor in PanNETs, these results show that in the setting of inactivated *Men1*, elevated TS efficiently accelerates the process of islet cell tumorigenesis. Consistent with these in vivo findings, we also showed that overexpression of hTS confers foci-forming activity in *Men1*-deleted MEF cells, implying that a pro-tumorigenic drive of overexpressed hTS in *Men1*-deleted genetic background occurs in both pancreatic islet cells and MEF cells. Since elevated levels of TS are frequently detected in PanNET patients (15, 37), our data suggest that high TS levels in inactivated *Men1*-null tissues might play an important role in PanNET progression.

The role of elevated hTS in altering cell cycle kinetics in PanNETs was demonstrated by loss of p21^Cip1^ and a reduction of p18^INK4c^ protein levels in PanNET tissues derived from *hTS/Men1*^+/*Δ*N3-8^ as compared with *Men1*^+/*Δ*N3-8^ mice. The importance of p21^Cip1^ and p18^INK4c^ in blocking islet cell growth was recently observed in a high-throughput functional genomics study, which identified CDK inhibitors as key factors blocking the proliferation of human β cells (38). p18^INK4c^ is known to inhibit the CDK4/CDK6 cyclin complex required for G_0_/G_1_ cell cycle progression (39), whereas p21^Cip1^ prevents G_1_ progression and S phase entry by blocking cyclin–CDK2/CDK4 cyclin complexes (40). Since expression of p21^Cip1^ and p18^INK4c^ is important for growth regulation of human islet cells, downregulation of these CDK inhibitors due to elevated TS levels may contribute to the progression of PanNETs. Tumors derived from *Men1*^+/*Δ*N3-8^ and *hTS/Men1*^+/*Δ*N3-8^ mice both showed LOH of the *Men1* WT copy; however, loss of p21^Cip1^ and reduction of p18^INK4c^ protein levels were observed only in tumors isolated from *hTS/Men1*^+/*Δ*N3-8^ but not from *Men1*^+/*Δ*N3-8^ mice. This observation suggests that inactivation of *Men1* through LOH was not sufficient to inhibit expression of CDK inhibitors and that overexpression of TS may have contributed to loss of p21^Cip1^ and reduction of p18^INK4c^ levels in PanNET tissues isolated from *hTS/Men1*^+/*Δ*N3-8^ mice. In contrast, immunochemistry performed on tumor tissue derived from *Men1^–/–^* mice demonstrated loss of p21^Cip1^ and p18^INK4c^ expression, suggesting that loss of menin may be sufficient for downregulation of p21^Cip1^ and p18^INK4c^ levels in *Men1^–/–^* mice. A possible explanation of this discrepancy may be that *Men1* LOH arises late in the life span of *Men1*^+/*Δ*N3-8^ mice while *Men1^–/–^* mice exhibit homozygous *Men1* deletion at birth resulting in accumulation of mutations needed for deregulation of p21^Cip1^ and p18^INK4c^ expression.

We also showed that overexpression of TS alone may be sufficient to downregulate p21^Cip1^ levels in MEF cells. This observation corroborates our published observation that overexpression of TS resulted in downregulation of both p21^Cip1^ protein and mRNA in MCF-7 cells (30). Deletion of *Men1* in MEF cells was not sufficient to downregulate p21^Cip1^ levels; however, overexpression of TS in *Men1*-null MEF cells resulted in downregulation of p21^Cip1^ protein. We also observed that ectopic TS overexpression in WT and in *Men1*-deleted MEFs did not alter p18^INK4c^ levels, whereas *Men1* deletion in MEF cells reduced the p18^INK4c^ level. Moreover, TS knockdown by shRNA in pancreatic neuroendocrine BON tumor cells upregulated p21^Cip1^ levels without affecting p18^INK4c^ protein expression. Taken together, our results show that overexpression of TS plays an important role in downregulation of p21^Cip1^ in vitro and in vivo while p18^INK4c^ level is predominantly regulated by the *Men1* gene product. Therefore, changes in the level of the cell cycle regulator p21^Cip1^ by high TS levels and changes of p18^INK4c^ level by deregulation of menin activity may trigger entry to G_1_/S phase and thus increase islet cell proliferation and tumor progression.

We also tested cell cycle kinetics and observed that elevated TS levels prolonged G_2_/M phase and were associated with DNA damage, resulting in enhanced chromosomal rearrangements in TS-overexpressing *Men1*-null MEF cells. The genomic instability induced by high levels of TS is not unexpected, because normal TS activity is essential for maintaining nucleotide homeostasis required for the error-free replication of the DNA (41, 42), and chromosomal rearrangements are frequently found in human PanNET samples (43–45). For example, increased expression of TS could result in nucleotide imbalance that may cause deoxythymidine triphosphate misincorporation into DNA, leading to DNA damage (46, 47). Consistent with this model, we also demonstrated increased somatic mutations with hTS overexpression in *hTS/Men1^–/–^*/BB mouse models that contained a CII reporter gene in BB mice as a biomarker of mutagenesis. CII genes recovered from 5-month-old *hTS/Men1^–/–^*/BB mice developed mutations including translocations, transitions, and deletions, while mutations were not observed in age-matched *Men1*^–/–^/BB mice. These somatic mutations observed in 5-month-old *hTS/Men1*^–/–^/BB mice due to TS overexpression correlate with the accelerated tumor progression found in age-matched *hTS/Men1^–/–^* mice.

We also analyzed the relationship between TS overexpression and concurrent mutations in either the *ATRX*, *DAXX*, or *MEN1* genes that represent the most frequently detected somatic mutations in human PanNETs. We observed that median TS gene expression in samples with *ATRX*, *DAXX*, or *MEN1* mutations was higher in comparison with samples carrying WT alleles of all 3 genes (*P* = 0.044) (Supplemental Figure 9). Since PanNET tumor databases are rarely annotated for TS expression, future prospective analyses will be required to determine whether TS levels are directly associated with enhanced mutational rates in PanNETs. To examine the relationship between TS overexpression and somatic mutations in non-PanNET samples, we analyzed TS expression in patients with 5 different tumor subtypes: prostate, pancreatic, lung adenocarcinoma, lung squamous carcinoma, and cutaneous melanoma, using data collected from The Cancer Genome Atlas (48) and the Clinical Proteomic Tumor Analysis Consortium. We observed a positive correlation between gene mutation frequency and elevated median TS transcript levels (Supplemental Figure 10). We also analyzed the correlation between median TS gene expression and the expression of a set of DNA damage response pathway genes using the PanNET gene expression data set (GSE117853, SubSeries GSE117851) deposited in GEO. We observed that TS gene expression positively correlated with 22 of the 79 genes (Supplemental Table 8). Further work will be required to define the association between TS expression and this subset of DNA damage–responsive genes in PanNETs.

In summary, TS overexpression is an important molecular event that underlies poor prognosis in many common adult tumor subtypes (13–19). In addition, current TS inhibitor anticancer agents, such as 5-fluorouracil, are associated with feedback induction of TS expression that is associated with drug resistance (49). We have now shown that elevated TS participates directly in promoting PanNET tumorigenesis using 3 different *Men1*-mutant animal models. These data emphasize the importance of development of a new class of TS inhibitors to block TS catalytic activity without feedback induction of TS levels. This work will refocus attention on new strategies to inhibit TS activity for PanNET treatment.

## Methods

### Mice.

*CMV-hTS* mice (designated *hTS* mice) (12), *RIP2-Cre/floxed Men1* mice (*Men1^–/–^* mice) (9), and heterozygous *Men1*-mutant mice (*Men1*^+/*Δ*N3-8^ mice) (8) were previously described. BB mice were purchased from Agilent Technologies. *Men1^–/–^* mice (B6;FVB;129/Sv mixed background) were crossed with *hTS* mice (FVB background) to generate *CMV-hTS/RIP2-Cre/floxed Men1* mice (designated *hTS/Men1^–/–^* and *hTS/Men1^+/–^* mice). Similarly, *Men1*^+/*Δ*N3-8^ mice (FVB;129S background) were crossed with *hTS* mice to generate *hTS/Men1*^+/*Δ*N3-8^ mice. Primer sequences for genotyping and histopathological assessment of tumor progression in mice tissues are provided in Supplemental Methods. BB mice (C57BL/6 background) were crossed with *Men1^–/–^* and *hTS/Men1^–/–^* mice to generate *Men1^–/–^*/BB and *hTS/Men1^–/–^*/BB mice. Mice were maintained within the University of Florida Cancer Genetics Research Center barrier facilities in individual ventilated cages. All animal experiments were done in accordance with IACUC-approved protocols. WT MEF cells (clone W10) and *Men1*-null MEF cells (clone N41.4) were derived from *Men1*^+/*Δ*N3-8^ mice and were a gift from Settara Chandresekharappa (NIH, Bethesda, Maryland, USA) (50). The details about establishment of TS-overexpressing clones from these MEF cells, cell viability assay, foci-forming assay, and cell cycle analysis conducted using TS-overexpressing MEF clones are provided in Supplemental Methods.

### Patients.

Patients with PanNETs who underwent surgery at Seoul National University Hospital were identified, and a total of 88 surgically resected pancreatic endocrine tumors were collected retrospectively. Age, sex, tumor location, hormonal function, angioinvasion, perineural invasion, and tumor size of patients were evaluated by review of medical charts and pathological records. Tissue slides were reviewed for WHO classification (51). Patient clinical outcome was followed up from the date of surgery in a period of 2 to 218 months.

### Immunohistochemistry.

Tissue sections (4 μm thickness) were cut from formalin-fixed paraffin-embedded tissue blocks, deparaffinized, and dehydrated. IHC staining was performed using the avidin-biotin complex method after antigen retrieval in a microwave. Information about antibodies used is provided in Supplemental Table 9. Immunostaining for p21^Cip1^, p18^INK4c^, and p27^Kip1^ was evaluated by a masked pathologist and quantified by the staining score, determined by multiplication of a staining intensity value (0, negative; 1, weak; 2, strong) by the percentage of tissue area stained (52). Mouse tonsil tissue and pancreas tissue were included as positive control, and negative controls were obtained by omission of the primary antibody for each immunostaining. For Ki-67 labeling index, the cell number was counted at ×400 original magnification using computerized image analyzer of ImageJ software. Only nuclear staining was regarded as positive, and the Ki-67 labeling index was calculated as the percentage in over 500 tumor cells. Details of processing and quantification of human tissues are described in Supplemental Methods.

### Comet assay.

DNA strand breaks were quantified by alkaline comet assay following the manufacturer’s protocol (4250-050-K, R&D Systems) as previously described (53) and reported according to the Minimum Information for Reporting Comet Assay recommendations (54). A detailed description of the comet assay is provided in Supplemental Methods.

More information regarding immunoblot analysis, BB mutation detection assay, metaphase preparation, spectral karyotyping analysis, γ-H2AX immunofluorescence, lentivirus production, and lentivirus-mediated TS inhibition in BON cells is provided in Supplemental Methods.

### Statistics.

Survival curves were analyzed by the Kaplan-Meier method, and the differences between groups were estimated by the Mantel log-rank test. Data are represented as mean ± SD or as mean ± SEM. Significance of differences between *hTS/Men1^–/–^* and *Men1^–/–^* mouse pancreas lesions was determined by the χ^2^ test, and differences in tumor incidence between *Men1*^ΔN3-8/+^ and *Men1*^ΔN3-8/+^ mice were analyzed by Fisher’s exact test. To determine significance in other experiments, we performed 2-tailed Student’s *t* test using Prism 9.0 (GraphPad Software) and SPSS 22.0 statistical software program (IBM). Significance of differences in cell proliferation was calculated by 2-way ANOVA followed by Tukey’s multiple comparisons. Significance for p21 and p18 densitometry quantification was calculated using 2-way ANOVA with multiple comparisons including the 2-stage linear step-up procedure of Benjamini, Krieger, and Yekutieli. Significance of differences in tail moment was calculated by Mann-Whitney *U* test. A *P* value less than 0.05 was considered statistically significant for all data sets.

### Study approval.

All animal experiments were done in accordance with protocols approved by the University of Florida Institutional Animal Care and Use Committee, according to national and institutional guidelines. All human studies were approved by the IRB for human subjects research of Seoul National University Hospital (approved IRB no. H-1602-034-739).

## Author contributions

MZK, VV, KM, and HSL designed the experiments. HSL, KM, VV, SB, KBL, BM, MWD, and AN performed the experiments. VV, KM, HSL, MVG, FJK, CJ, and MZK analyzed and interpreted the data. HSL and KBL provided the patient samples and histology interpretation. IM and TJG performed bioinformatics analysis. MF analyzed pathology slides. MZK, VV, KM, and FJK wrote the manuscript. All authors reviewed the manuscript.

## Supplementary Material

Supplemental data

## Version 1 09/01/2022

In-Press Preview

## Version 2 10/10/2022

Electronic publication

## References

[1] Dasari A (2017). Trends in the incidence, prevalence, and survival outcomes in patients with neuroendocrine tumors in the United States. JAMA Oncol.

[2] Halfdanarson TR (2008). Pancreatic neuroendocrine tumors (PNETs): incidence, prognosis and recent trend toward improved survival. Ann Oncol.

[3] Scarpa A (2017). Whole-genome landscape of pancreatic neuroendocrine tumours. Nature.

[4] Sadanandam A (2015). A cross-species analysis in pancreatic neuroendocrine tumors reveals molecular subtypes with distinctive clinical, metastatic, developmental, and metabolic characteristics. Cancer Discov.

[5] Mafficini A, Scarpa A (2018). Genomic landscape of pancreatic neuroendocrine tumours: the International Cancer Genome Consortium. J Endocrinol.

[6] Jiao Y (2011). DAXX/ATRX, MEN1, and mTOR pathway genes are frequently altered in pancreatic neuroendocrine tumors. Science.

[7] Matkar S (2013). Menin: a scaffold protein that controls gene expression and cell signaling. Trends Biochem Sci.

[8] Crabtree JS (2001). A mouse model of multiple endocrine neoplasia, type 1, develops multiple endocrine tumors. Proc Natl Acad Sci U S A.

[9] Crabtree JS (2003). Of mice and MEN1: insulinomas in a conditional mouse knockout. Mol Cell Biol.

[10] Wong C (2020). Two well-differentiated pancreatic neuroendocrine tumor mouse models. Cell Death Differ.

[11] Locasale JW (2013). Serine, glycine and one-carbon units: cancer metabolism in full circle. Nat Rev Cancer.

[12] Chen M (2007). Transgenic expression of human thymidylate synthase accelerates the development of hyperplasia and tumors in the endocrine pancreas. Oncogene.

[13] Wilson PM (2014). Standing the test of time: targeting thymidylate biosynthesis in cancer therapy. Nat Rev Clin Oncol.

[14] Monica V (2009). Differential thymidylate synthase expression in different variants of large-cell carcinoma of the lung. Clin Cancer Res.

[15] Lee HS (2014). Analysis of 320 gastroenteropancreatic neuroendocrine tumors identifies TS expression as independent biomarker for survival. Int J Cancer.

[16] Allegra C (2002). Thymidylate synthase levels: prognostic, predictive, or both?. J Clin Oncol.

[17] Hu YC (2003). Thymidylate synthase expression predicts the response to 5-fluorouracil-based adjuvant therapy in pancreatic cancer. Clin Cancer Res.

[18] Bertino JR, Banerjee D (2003). Is the measurement of thymidylate synthase to determine suitability for treatment with 5-fluoropyrimidines ready for prime time?. Clin Cancer Res.

[19] DiPaolo A, Chu E (2004). The role of thymidylate synthase as a molecular biomarker. Clin Cancer Res.

[20] Rahman L (2004). Thymidylate synthase as an oncogene: a novel role for an essential DNA synthesis enzyme. Cancer Cell.

[21] Schaller B (2003). Gender-related differences in non-functioning pituitary adenomas. Neuro Endocrinol Lett.

[23] Bertolino P (2003). Heterozygous Men1 mutant mice develop a range of endocrine tumors mimicking multiple endocrine neoplasia type 1. Mol Endocrinol.

[24] Scacheri PC (2004). Pancreatic insulinomas in multiple endocrine neoplasia, type I knockout mice can develop in the absence of chromosome instability or microsatellite instability. Cancer Res.

[25] Wander SA (2011). p27: a barometer of signaling deregulation and potential predictor of response to targeted therapies. Clin Cancer Res.

[26] Park BJ (2005). The haploinsufficient tumor suppressor p18 upregulates p53 via interactions with ATM/ATR. Cell.

[27] Fero ML (1998). The murine gene p27Kip1 is haplo-insufficient for tumour suppression. Nature.

[28] El-Deiry WS (2016). p21(WAF1) mediates cell-cycle inhibition, relevant to cancer suppression and therapy. Cancer Res.

[29] Karnik SK (2005). Menin regulates pancreatic islet growth by promoting histone methylation and expression of genes encoding p27Kip1 and p18INK4c. Proc Natl Acad Sci U S A.

[30] Kastanos EK (2001). Downregulation of p21/WAF1 expression by thymidylate synthase. Biochem Biophys Res Commun.

[31] Schnepp RW (2006). Mutation of tumor suppressor gene Men1 acutely enhances proliferation of pancreatic islet cells. Cancer Res.

[32] Kastan MB, Bartek J (2004). Cell-cycle checkpoints and cancer. Nature.

[33] Jeggo PA, Lobrich M (2007). DNA double-strand breaks: their cellular and clinical impact?. Oncogene.

[34] Chon J (2017). Targeting nuclear thymidylate biosynthesis. Mol Aspects Med.

[35] https://www.R-project.org/.

[36] Zhang J (2013). Current understanding of the molecular biology of pancreatic neuroendocrine tumors. J Natl Cancer Inst.

[37] Ceppi P (2008). Thymidylate synthase expression in gastroenteropancreatic and pulmonary neuroendocrine tumors. Clin Cancer Res.

[38] Robitaille K (2016). High-throughput functional genomics identifies regulators of primary human beta cell proliferation. J Biol Chem.

[39] Sherr CJ, Roberts JM (1995). Inhibitors of mammalian G1 cyclin-dependent kinases. Genes Dev.

[40] Georgakilas AG (2017). p21: a two-faced genome guardian. Trends Mol Med.

[41] Ser Z (2016). Targeting one carbon metabolism with an antimetabolite disrupts pyrimidine homeostasis and induces nucleotide overflow. Cell Rep.

[42] Voeller D (2004). Elevated levels of thymidylate synthase linked to neoplastic transformation of mammalian cells. Cell Cycle.

[43] Gebauer N (2014). Genomic landscape of pancreatic neuroendocrine tumors. World J Gastroenterol.

[44] Lawrence B (2018). Recurrent loss of heterozygosity correlates with clinical outcome in pancreatic neuroendocrine cancer. NPJ Genom Med.

[45] Williamson LM (2019). Genomic characterization of a well-differentiated grade 3 pancreatic neuroendocrine tumor. Cold Spring Harb Mol Case Stud.

[46] Aird KM, Zhang R (2015). Nucleotide metabolism, oncogene-induced senescence and cancer. Cancer Lett.

[47] Mathews CK (2006). DNA precursor metabolism and genomic stability. FASEB J.

[48] Cancer Genome Atlas Research Network (2013). The Cancer Genome Atlas Pan-Cancer analysis project. Nat Genet.

[49] Crea F (2011). Epigenetics and chemoresistance in colorectal cancer: an opportunity for treatment tailoring and novel therapeutic strategies. Drug Resist Updat.

[50] Ji Y (2007). Mouse embryo fibroblasts lacking the tumor suppressor menin show altered expression of extracellular matrix protein genes. Mol Cancer Res.

[52] Yamaguchi Y (2010). Histopathologic features of the tumor budding in adenocarcinoma of the lung: tumor budding as an index to predict the potential aggressiveness. J Thorac Oncol.

[53] He Z (2019). Campylobacter jejuni promotes colorectal tumorigenesis through the action of cytolethal distending toxin. Gut.

[54] Moller P (2020). Minimum information for reporting on the comet assay (MIRCA): recommendations for describing comet assay procedures and results. Nat Protoc.

