# Supplementary material for: Thymidylate synthase accelerates Men1-mediated pancreatic tumor progression and reduces survival
Source: JCI Insight. 2022 Oct 10;7(19):e147417. doi: 10.1172/jci.insight.147417 (PMC9675466; doi:10.1172/jci.insight.147417)
Supplement: Supplemental data [file jciinsight-7-147417-s073.pdf]

Supplemental Methods

Mice genotyping

Mice were genotyped using the following primers as previously described (1-3): 8F, 5’-GCCATTTCATTACCTCTTTCTCCG-3’ and 8R, 5’-TACCACTGCAAAGGCCACGC-3’ to demonstrate loss of the floxed *Men1*; NCI2 5'- ATGCCCTCTGCCAGTTCTATGTGG-3' and H2I 5'-TAGAAGGCACAGTCGAGG-3', detect human *TS*; 5’-CCGGGCTGCCACGACCAA-3’ and 5’-GGCGCGGCAACACCATTTTT-3’ to detect RIP2-*Cre*. *Men1* wild type and $Men1^{+/\Delta N3–8}$ mice were genotyped with a multiplex PCR primers, A, B, and D. A: 3'-CCCACATCCAGTCCCTCTTCAGCT-5', B: 5'-CCCTCTGGCTATTCAATGGCAGGG-3', D: 5'-CATAAAATCGCAGCAGGTGGGCAA-3'. Combination of primers A and B yields wild-type *Men1* PCR product of 300 bp, and combination of primers A and D yields a 638 bp ΔN3–8-specific *Men1* PCR product. Primer *A* is located in exon 2 of *Men1* gene, primer B is specific for an exon 3 of *Men1* gene and primer D is located in exon 9 of *Men1* gene. Primers for PCR amplification and sequencing of CII gene are cII-F 5´-CCACACCTATGGTGTATG-3´ and cII-R 5´-CCTCTGCCGAAGTTGAGTAT-3´.

Histopathology of Genetically Engineered Mouse Models

After euthanasia, tissues were collected and snap frozen for protein and mRNA extraction. The remaining tissue was placed in a biopsy cassette for histopathological examination. Tissue was then fixed in 10% formalin, embedded in paraffin, sectioned, and stained with H&E. Pancreatic tissues were scored as normal, hyperplasia, adenoma, and carcinoma by masked pathologists according to criteria previously described (4).

Islet hyperplasia was scored when multiple islets were greater than 100 microns and had normal architecture. For islet cell adenoma, architecture was similar to hyperplasia, but size of islet was larger with compression of adjacent acinar pancreas and more prominent vascular dilation. Islet cell carcinoma showed capsular invasion and increased cellular pleomorphism, fibrosis and mitotic figures.

Establishment of stable clones of MEF cells with hTS overexpression

MEF wild type (clone W10) and MEF-*Men1*-null (clone N41.4) cell lines were obtained from Dr. Chandresekharappa (NIH) (5) and were cultured in Dulbecco's modified Eagle's medium (DMEM) supplemented with 10% fetal bovine serum, penicillin (100 U/mL) and streptomycin (100 µg/mL) at 37°C under 5% CO_2. To generate stable cell lines overexpressing TS, TS cDNA was cloned into a retroviral expression vector, pLNCX (Clontech) resulting in pLNCX-TS. To construct the pLNCX-TS expression vector, a 1965 bp fragment of TS cDNA plus 3'UTR was excised from pOTB7-TS using *ScaI* and *SmaI* sites and the fragment was subcloned into *HpaI* site in pLNCX vector. To generate retrovirus, HEK293T cells were transfected with 10 µg of pLNCX-TS or pLNCX empty vector, and 2.5 µg retroviral packaging plasmids (pMD-MuLV and pMD-G, Addgene) using Effectene (Qiagen Cat# 301427) as per the manufacturer's instructions. Briefly, the transfection mixture was prepared by adding pLNCX-TS or pLNCX plasmid, the enhancer reagent provided in the Qiagen kit (16 µl) and Effectene (60 µl) to EC buffer (300 µl) and incubated for 10 min, followed by addition of 3 ml of 10% DMEM to the mix. HEK293T cells were seeded in a T75 flask at a density of 2 x 10^6 cells and the retroviral mixture was added 24h after plating. Transfection mixture was removed from the flask after 8h, replaced with fresh medium and cells were incubated for an additional 48h. Media was

then collected and spun at 3000 g for 3 min to remove residual HEK293T cells and the supernatant was transferred to a new tube and filtered through a 0.45 μm syringe filter. The virus preparation was kept at -80°C until use.

For transduction, 1 x 10^6 MEF wild type and MEF-*Men1*$^{-/-}$ cells were seeded in 100-mm dishes, then media was removed after 24h and plates were incubated with 4 ml of viral supernatant (containing 2 μg /ml of polybrene (Millipore) for 24h to transduce cells with retroviral TS cDNA or vector control (pLNCX-TS and pLNCX) (6). After 24h, virus containing media was removed and replaced with selection media (DMEM supplemented with 10% FBS, 1% Penicillin-Streptomycin and G418 [300 μg/ml]). Cells were incubated with selection media for 2 weeks, with media change every two days, to select single cell clones that stably overexpress TS or control vector (designated as MEF-V, MEF-V/*Men1*$^{-/-}$, MEF-TS and MEF-TS/*Men1*$^{-/-}$). Selected single cell clones for each assay are described in detail below. After expansion and harvest, TS expression was determined by immunoblot analysis.

Cell viability assay

Viability of cells was measured using MTS reagent as recommended by manufacturer instructions (Promega). Distinct clones of MEF-TS, MEF-TS/*Men1*$^{-/-}$ cells and their corresponding control vector expressing cells were seeded in 96-well plates (1 x 10^3 cells/well; each clone was seeded into six wells in a 96 well plate). Plates were incubated for 24, 48 and 72 hours. At each time point, 10 μl of MTS reagent was added to each well without removing the media, incubated at 37°C for 1 hour and assayed using a micro plate reader at 490 nm wavelength (SpectraMax M3, Molecular Devices).

Three clones of MEF-TS cells (TS1-1, TS1-2 and TS1-3) and two clones of MEF-V cells (V1-1 and V1-2) were tested in duplicate, and the average proliferation rate was analyzed at different time points. In addition, three clones of MEF-TS/*Men1*$^{-/-}$ cells (TS2-1, TS2-2 and TS2-4) and two clones of MEF-V/*Men1*$^{-/-}$ cells (V2-1 and V2-2) were tested in duplicate in two independent experiments and the average proliferation rate for TS2-1, TS2-2, TS2-4 and V2-1 and V2-2 was combined and analyzed at different time points.

Foci forming assay

MEF-TS (TS1-1), MEF-TS/*Men1*$^{-/-}$ (TS2-1) cell clones and their corresponding vector-expressing cell clones (V1-1 and V2-1, respectively) were seeded in 100 mm petri dishes (1 x 10^5 cells/plate) and were grown in 10% DMEM with G418 (300 µg/ml). Cells were incubated for 3 weeks, with media changed every 3 to 4 days. Cells were fixed using 10% methanol and 10% acetic acid fixing solution and stained with 20% ethanol and 0.4% crystal violet, for 5 min.

Cell cycle analysis

Cell cycle analysis was performed by flow cytometry (FACSort; Becton Dickinson). Cells (1 x 10^6) were harvested, washed 2x with PBS and suspended in 200 ul of PBS. Cells were fixed using 4 ml of cold 75% ethanol at 4°C overnight and then washed 2x with PBS. Cells were then suspended in 500 ul of PBS, stained by addition of 200 µl of propidium iodide (50 µg/ml, Sigma) and RNase A (0.5 µg/mL, Sigma) for 10 min in the dark at room temperature. 1x10^4 cells were measured per sample, and the analysis was performed using CellQuest Pro software (Becton Dickinson). Flow cytometry procedures were performed at the Interdisciplinary Center for Biotechnology Research (ICBR) Cytometry

Core Facility at University of Florida. For cell cycle analysis we compared 3 sets of clones: 1) MEF-V vs. MEF-V/*Men1*$^{-/-}$, 2) MEF-V vs. MEF-TS and 3) MEF-V/*Men1*$^{-/-}$ vs. MEF-TS/*Men1*$^{-/-}$. Two different clones were used for each cell type and all experiment were repeated 2 to 4 times as shown below. n = number of experiments performed for each clone. MEF-V clones V1-1 (n = 4) and V1-2 (n = 4) vs. MEF-V/*Men1*$^{-/-}$ clones V2-1 (n = 2) and V2-2 (n = 3). MEF-TS (TS1-1 n = 3 and TS1-2 (n = 3) vs. MEF-V (V1-1 n = 4 and V1-2 n=4). MEF-TS/*Men1*$^{-/-}$ (TS2-1 n = 4 and TS2-2 n = 3) vs. MEF-V/*Men1*$^{-/-}$ (V2-1 n = 2 and V2-2 n = 3).

Immunohistochemistry of human tissue

Immunohistochemical analysis of TS, ɣ-H2AX and Ki-67 in human patient samples was performed using three tissue array blocks containing 88 human pancreatic neuroendocrine tumors prepared as described previously (7). Briefly, core tissue biopsies (2 mm in diameter) were taken from individual paraffin-embedded tumors (donor blocks) and arranged in recipient paraffin blocks (tissue array blocks) using a trephine. A representative core was sampled in each tumor tissue and an adequate case was defined as a tumor occupying more than 10% of the core area. Four μm sections were cut from each tissue array block, deparaffinized and dehydrated. Immunohistochemical staining for TS (1:100, Fisher Scientific Cat# MAB4130MI) ɣ-H2AX (1:300, Upstate Cat# 05-636) and Ki-67 (1:500, Abcam Cat# ab92742) was performed using an automated immunostainer (Ventana BenchMark XT, Ventana Medical System, Tucson AZ, USA). Staining intensity and stained tumor cell percentage of TS were measured as previously described (7). Staining intensity was classified as negative, weak and strong expression. For statistical analyses, the staining was considered as positive when present in more

than 5% of the tumor cell population. For ɣ-H2AX and Ki-67 immunohistochemistry stained slides were scanned using an Aperio AT2 scanner (Leica Biosystem, Newcastle upon Tyne, UK), then percentage of ɣ-H2AX and Ki-67 positive cells were evaluated using QuPath (open-source software) (8).

Comet Assay

MEF cells were cultured for 48 h before being collected using trypsin and resuspended in low-melting agarose (R&D Systems) at a final concentration of 0.9% before spreading on 20-well Comet Slides (R&D Systems). Comet slides were incubated in lysis buffer (R&D Systems) for 1 h, at 4°C to lyse cells before washing twice with deionized H_2O and submerging Comet Slides in an alkaline electrophoresis solution (deionized H_2O containing 200 mM NaOH and 1 mM EDTA, pH > 13) for 20 minutes at room temperature. Electrophoresis was run for 30 min with applied voltage of 21 V and a current of approximately 300 mA using the Comet Assay Electrophoresis System II (R&D Systems). The electrophoresis unit and buffer were chilled to 4°C. Slides were rinsed twice in deionized H_2O, fixed in ethanol for 5 minutes, and dried for 10 minutes at 37°C using a standard incubator. DNA was stained with 40 µl 1x SYBR Gold nucleic acid gel stain (S-11494, Life Technologies) per well and immediately visualized using the Leica DM6000B upright microscope. DNA damage was quantified using the OpenComet Image-J plugin (9). Data points, shown as black dots in the bar diagram, represent mean tail moment of at least 10 cells of a MEF clone from a single comet experiment. The number of cells quantified for each data point are as follows: MEF-V (V1-1: 20 and 35 cells; V1-2: 48 and 15 cells), MEF-TS (TS-1: 12 and 23 cells; TS1-2: 29 and 11 cells; TS1-3: 62 and 20 cells),

MEF-V/*Men1*$^{-/-}$ (V2-1: 27 and 20 cells; V2-3: 22, 25 and 33 cells) and MEF-TS/*Men1*$^{-/-}$ (TS2-1: 38, 31 and 20 cells; TS2-3: 47 and 13 cells).

Immunoblot analysis

Tissues were homogenized using the Omni general laboratory homogenizer (GLH) following the manufacturer's protocol (OMNI international). Cell lines were collected by scraping and washed 2x with cold PBS. Cells and tissue homogenates were lysed in a RIPA lysis buffer (Santa-Cruz, sc-24948) and centrifuged (12000 rpm, 20 min) to obtain whole-cell lysates. Protein concentration was measured using the Bradford assay. Aliquots of the lysates (10-20 µg of protein) were separated on 10% or 4-20% Tris-glycine gels, transferred to nitrocellulose membranes, and probed with specific antibodies (Supplemental Table 9); Blots were visualized by Super Signal Chemiluminescent Dura substrate (ThermoFisher Scientific). For densitometric analysis of proteins, Image-J software was used for each immunoblot to quantify band intensity, and then each band intensity value was normalized to loading control.

Big Blue® mutation detection assay

Men1$^{-/-}$/BB and *hTS*/*Men1*$^{-/-}$/BB mice carry bacteriophage lambda shuttle vector (LIZ) containing CII gene as the target of mutagenesis. The mutation detection assay was carried out using the Transpak® and the λ Select-cII™ protocol (Agilent Technology). Genomic DNA was isolated from mice pancreas at 5 months and 10 months of age. Genomic DNA was packaged *in vitro* to produce virulent lambda phage, infecting *E. coli* strain G1250 and plated. Plates were incubated under mutant selective (24°C, 40 h) or

control (37°C, 24 h) conditions. Approximately 300,000 ~ 800,000 plaque forming units (PFUs) were analyzed from multiple sample preparations of pancreas tissue or pancreas tumors of age matched groups of mice. The mutation frequency was calculated as the ratio of the number of mutant plaques (grown at 24°C, 40 h) per total number of plaques (grow at 37°C, 24 h). Normalization of mutation frequency was calculated by subtracting the false positive plaques derived from 5 months old *Men1*$^{-/-}$/BB mice. 195 mutant plaques were isolated using sterile pipette tips, PCR amplified and sequenced to further verify the type of mutations present in the sequence. Primers for PCR amplification and sequencing of CII gene are cII-F 5´-CCACACCTATGGTGTATG-3´ and cII-R 5´-CCTCTGCCGAAGTTGAGTAT-3´.

Metaphase preparation and spectral Karyotype analysis (SKY)

For chromosome aberration analysis, MEF-V/*Men1*$^{-/-}$ and MEF-TS/*Men1*$^{-/-}$ cells (0.5 x 10^6 cells) were seeded in a 100 mm dish, and incubated for 48h. Cells were blocked in metaphase by treatment with 0.01 mg/ml colcemid in HBSS (Karyomax, Invitrogen) at 37°C for 3 h and were further harvested by trypsinization, and pelleted by centrifugation (1000 rpm for 10 min). Cell pellets were suspended in 3 ml of hypotonic solution (0.075 M potassium chloride) and incubated at 37°C for 10 min. The cells were pelleted further by centrifugation and resuspended in 5 ml of 3:1 methanol/acetic acid for fixation. Metaphase slides were prepared by dropping 20 µl of fixed cell suspension onto a clean slide and incubated at 42°C overnight. Metaphases were hybridized with the 20-color mouse SKY paint kit according to manufacturer's protocol (Applied Spectral Imaging Inc). Spectral images of the hybridized metaphases were acquired using a SD301 SpectraCubeTM system (Applied Spectral Imaging Inc) mounted on top of an

epifluorescence microscope Axioplan 2 (Zeiss). Images were analyzed using Spectral Imaging 4.0 acquisition software (Applied Spectral Imaging Inc). G banding was simulated by electronic inversion of DAPI-counterstaining. Spectral karyotype analysis procedure was performed at the Comparative Molecular Cytogenetics Core Facility at NCI-Frederick.

Immunofluorescence analysis for **γH2AX staining**

MEF-TS (TS1-1), MEF-TS/*Men1*$^{-/-}$ cells (TS2-1) and their corresponding vector control cells (clones V1-1 and V2-1, respectively) were plated in a coverslip placed in 6 well plate (1 x 10^6 cells per well) and incubated for 24h. Cells were fixed in 4% paraformaldehyde for 10 min. After fixation, cells were permeabilized with 0.5% Triton X100 diluted in PBS and then blocked with 10% FBS in phosphate saline buffer (PBS) for 30 min. Cells were incubated with primary antibodies against γH2AX for 1 h at room temperature and with Alexa Fluor-conjugated secondary antibodies. The cells were mounted using VectaShield hard-set mounting media containing DAPI (Vector laboratories). Fluorescent images were recorded using an Olympus DP70 digital camera coupled to an Olympus IX71 inverted microscope (Olympus Corp). Percent fluorescence expression efficiency was measured using Image-J software.

Lentivirus production and lentivirus-mediated TS inhibition in BON cells

To produce lentiviral TS shRNA for transduction, HEK293T cells (12 x 10^6) were seeded in T175 flask and incubated overnight at 37ºC and 5% CO_2 for 10-20h. Cells were transfected with either 16 µg of TS shRNA encoding plasmid, (#60 Sigma, TRCN0000045663), (#61 Sigma, TRCN0000045664), (#64 Sigma, TRCN0000045667),

(#133 Dharmacon, RHS4430-200162272), or Non-specific (NS) shRNA (#71 Sigma, SHC202), (#128 Dharmacon, RHS4346). shRNA plasmids were co-transfected with 8 µg psPAX (lentiviral packaging plasmid) and 8 µg pMD2.G (VSV-G envelope expressing plasmid) using a mixture of 1 mg/ml polyethyleneimine (Polysciences) and 1.5 M NaCl. Viral supernatant was collected, filtered through a 0.45 µm filter 40h post-transfection and pooled together. Pooled supernatants were concentrated using an Amicon Ultra-15 100K cutoff filter device (EMD Millipore) according to manufacturer's instructions. Final concentrated virus was aliquoted and stored at -80ºC until used. For transient transduction, BON cells (5 x 10^6), gift from Kirk Ives (University of Texas Medical Branch, Galveston, TX), were plated in 100 mm dishes and incubated with the media containing lentiviral packaged TS shRNA or NS shRNA (containing TS shRNA) and 8 µg/mL polybrene (Millipore, TR-1003-G) for 16 h. After incubation with lentivirus, the cells were used either for western blotting to validate transduction or seeded in a 96 well plate for cell viability assay (MTS assay). For stable transduction, BON cells (5 x 10^6) were seeded in a 6 well plate and incubated overnight, then the cells were incubated with media containing lentiviral packaged with either TS shRNA # 133 or NS shRNA # 128 along with 8 µg/mL polybrene (Millipore, TR-1003-G). The plate was then centrifuged at 500 rpm for 10 minutes, incubated for 6 h, then the virus containing media was replaced with growth media followed by overnight incubation. This procedure of transduction was repeated on the same cells three times. After the final transduction, cells were incubated for 48 h in antibiotic free media. At 80% confluence, cells were split into 60 mm dishes and maintained in selection media containing 4 µ/mL puromycin. Stable transduction of TS shRNA in BON cells was verified by GFP positivity and decreased TS expression by immunoblotting.

Immunohistochemistry of chromogranin A and synaptophysin

Immunostaining was performed in paraffin-embedded tissues. In brief, 4 µm sections were de-paraffinized, then treated with Citra Steam (Biogenex) for 30 minutes. Background Sniper (Biocare Medical) was used for 15 minutes to reduce unspecific background staining. Sections were incubated with rabbit anti mouse/human chromogranin A (1:200, Abcam # 15160) or rabbit anti mouse/rat/human synaptophysin (1:1000, Abcam # 68851) for 60 minutes. Stain was visualized using Mach 2 rabbit HRP polymer (Biocare Medical), and the DAB chromogen (Vector Laboratories) with CAT hematoxylin counterstain (Biocare Medical).

Ultra-High-Pressure Liquid Chromatography coupled High-Resolution Mass Spectrometry (UHPLC-HRMS)-based metabolomics data collection and analysis

MEF-V (clone V1-1), MEF-TS (clone TS1-1), MEF-V/*Men1*$^{-/-}$ (clone V2-1) and MEF-TS/*Men1*$^{-/-}$ (clone TS2-5) were used for the metabolomics data analysis. UHPLC-HRMS-based untargeted metabolomics was used for data collection. Chromatographic separation for metabolomics was achieved using reversed-phase chromatography with a C18-pfp column (Ace, Aberdeen, Scotland; 100 x 2.1 mm, 2 µm). The mobile phases consisted of solvent A (0.1% formic acid in H_2O) and solvent B (acetonitrile). The system was held constant from 0-3 min at 100% A, then mobile phase B was ramped from 0% B to 80% over 10.0 min (3-13 min) and then held constant at 80% B for 3 min (13-16 min) with a flow rate of 350 µL/min and column temperature of 25°C. For equilibration, the system was returned to initial conditions with 0% B and the flow rate was increased to 600 µL/min. The flow rate was reduced back to 350 µL/min before the next injection. The

data collection time per sample was 20.5 min. Both positive (injection volume 2 μL) and negative ion polarity (injection volume 3 μL) in full scan mode (35,000 mass resolution) were acquired.

For metabolomics data analysis, metabolite identification was performed with MZmine 2.0 and matching metabolite retention time and m/z value to an internal library of over 1000 metabolites representing level 1 identification following metabolomics standards initiative guidelines. Metabolic pathway analysis was conducted as previously mentioned (10). Briefly, metabolic pathway analysis was conducted using the Kyoto Encyclopedia of Genes and Genomes (KEGG) pathway database by matching metabolite sets with human metabolome (https://www.genome.jp/kegg/pathway.html). Metabolite set enrichment (fold enrichment) was further investigated using MetaboAnalyst (open-source R package). Pathway impact score was computed from pathway topological analysis using relative-betweenness centrality.

Secondary analysis of data sets from patient tumor samples

PanNET transcriptomic patient data was extracted from GSE117853 (subseries: GSE117851). List of genes related to DNA damage response was obtained from https://www.mdanderson.org/documents/Labs/Wood-Laboratory/human-dna-repair-genes.html. Correlation between TS expression and other genes related to nucleotide synthesis as well as DNA damage was analyzed in Graph Pad Prism. Pearson correlation between -1 and 1, with p value ≤ 0.05 was considered statistically significant. Data to study TS gene expression in patient samples with mutations in *ATRX*, *DAXX*, or *MEN1* genes was obtained from GSE117853 (subseries: GSE117851).

To study the correlation between TS expression and mutation rate in patient samples of different tumor origin, we determined TS expression in human patients from 6 tumor types included in The Cancer Genome Atlas (11): prostate adenocarcinoma (PRAD, n = 497), pancreatic adenocarcinoma (PAAD, n = 178), lung adenocarcinoma (LUAD, n = 515), lung squamous cell carcinoma (LUSC, n = 501) and skin cutaneous melanoma (SKCM, n = 103). As shown in Supplemental Figure 10, tumors were organized by median TS expression that varied across tumor types from prostate adenocarcinoma (lowest TS expression) to skin cutaneous melanoma (highest TS expression). Processed data tables are available at LinkedOmics (http://www.linkedomics.org) (12). As part of our study, we compared the TS expression in different tumor types with the somatic mutation frequency data of various tumor types obtained from Lawrence *et al.* 2013 (13).

Supplemental references

1. Chen M, et al. Transgenic expression of human thymidylate synthase accelerates the development of hyperplasia and tumors in the endocrine pancreas. *Oncogene.* 2007;26(33):4817-4824.
2. Crabtree JS, et al. A mouse model of multiple endocrine neoplasia, type 1, develops multiple endocrine tumors. *Proc Natl Acad Sci U S A.* 2001;98(3):1118-1123.
3. Crabtree JS, et al. Of mice and MEN1: Insulinomas in a conditional mouse knockout. *Mol Cell Biol.* 2003;23(17):6075-6085.
4. Capen CC, et al. In: Mohr U ed. *International Classification of Rodent Tumors The Mouse* Springer; 2001:269-322.

5. Ji Y, et al. Mouse embryo fibroblasts lacking the tumor suppressor menin show altered expression of extracellular matrix protein genes. *Mol Cancer Res.* 2007;5(10):1041-1051.

6. Yang G, et al. Silencing of H-ras gene expression by retrovirus-mediated siRNA decreases transformation efficiency and tumorgrowth in a model of human ovarian cancer. *Oncogene.* 2003;22(36):5694-5701.

7. Lee HS, et al. Analysis of 320 gastroenteropancreatic neuroendocrine tumors identifies TS expression as independent biomarker for survival. *Int J Cancer.* 2014;135(1):128-137.

8. Bankhead P, et al. QuPath: Open source software for digital pathology image analysis. *Sci Rep.* 2017;7(1):16878.

9. Gyori BM, et al. OpenComet: an automated tool for comet assay image analysis. *Redox Biol.* 2014;2:457-465.

10. Lee B, et al. Medulloblastoma cerebrospinal fluid reveals metabolites and lipids indicative of hypoxia and cancer-specific RNAs. *Acta Neuropathol Commun.* 2022;10(1):25.

11. Cancer Genome Atlas Research N, et al. The Cancer Genome Atlas Pan-Cancer analysis project. *Nat Genet.* 2013;45(10):1113-1120.

12. Vasaikar SV, et al. LinkedOmics: analyzing multi-omics data within and across 32 cancer types. *Nucleic Acids Res.* 2018;46(D1):D956-D963.

13. Lawrence MS, et al. Mutational heterogeneity in cancer and the search for new cancer-associated genes. *Nature.* 2013;499(7457):214-218.

14. Rahman L, et al. Thymidylate synthase as an oncogene: a novel role for an essential DNA synthesis enzyme. *Cancer Cell.* 2004;5(4):341-351.

Supplemental Figure 1

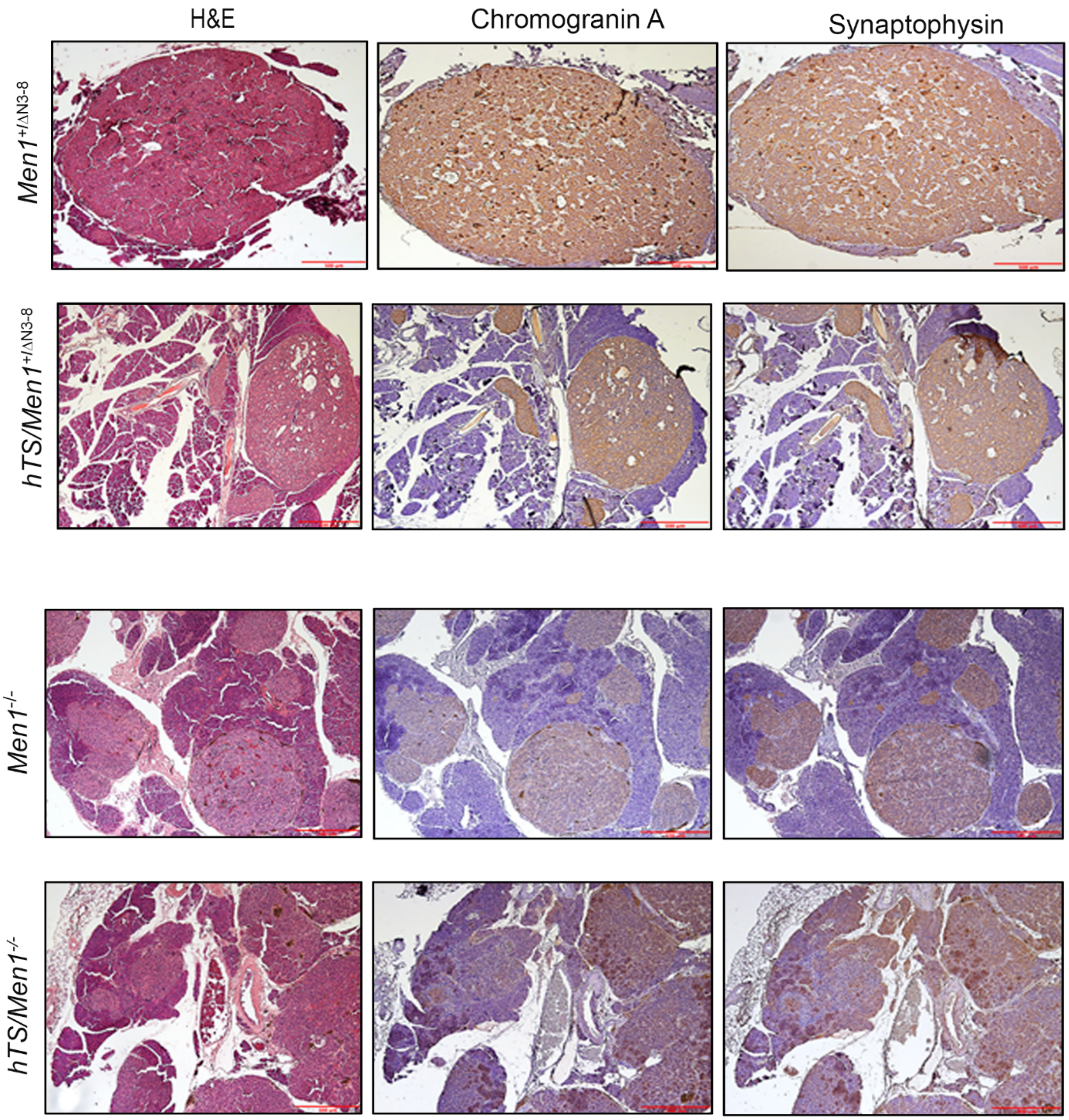

Supplemental Figure 1. Expression of chromogranin A and synaptophysin in tumor developed in *Men1*$^{+/\Delta N3\text{-}8}$, *hTS/Men1*$^{+/\Delta N3\text{-}8}$, *Men1*$^{-/-}$, and *hTS/Men1*$^{-/-}$ mice. H&E and immunohistochemical staining of chromogranin A and synaptophysin in PanNET developed in *Men1*$^{+/\Delta N3\text{-}8}$, *hTS/Men1*$^{+/\Delta N3\text{-}8}$, *Men1*$^{-/-}$, *and hTS/Men1*$^{-/-}$ *mice* mouse models. Scale bars, 50 μm.

Supplemental Figure 2

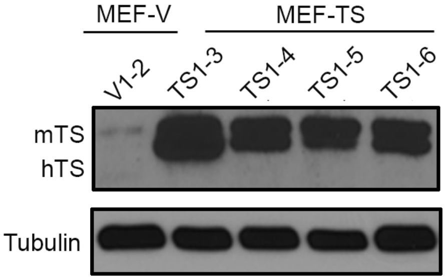

Supplemental Figure 2. Ectopic overexpression of hTS in MEF cells. Immunoblot analysis of human TS (hTS) and mouse TS (mTS) in distinct MEF cells clones transfected with hTS expressing plasmid (TS) or empty vector (V). Tubulin is used as loading control.

Supplemental Figure 3

A

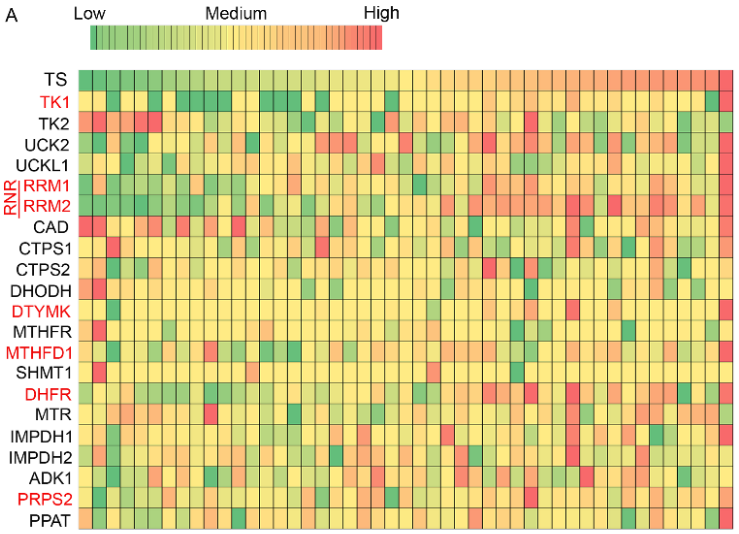

B

Genes	Correlation with TS, pearson(R)	p-value
TK1	0.4058	0.0047
TK2	-0.4892	0.0005
UCK2	0.3634	0.012
UCKL1	0.2535	0.0855
RRM1	0.4720	0.0008
RRM2	0.7431	<0.0001
CAD	-0.2396	0.1048
CTPS1	-0.8770	-0.5577
CTPS2	0.0016	0.9915
DHODH	-0.3814	0.0082
DTYMK	0.3434	0.0181
MTHFR	-0.0374	0.0096
MTHFD1	0.3472	0.0168
SHMT1	-0.2477	0.0932
DHFR	0.4819	0.0006
MTR	-0.0337	0.8218
IMPDH1	0.2065	0.1637
IMPDH2	0.0449	0.7644
ADK1	0.1882	0.2052
PRPS2	0.4369	0.0021
PPAT	0.1858	0.2112

C

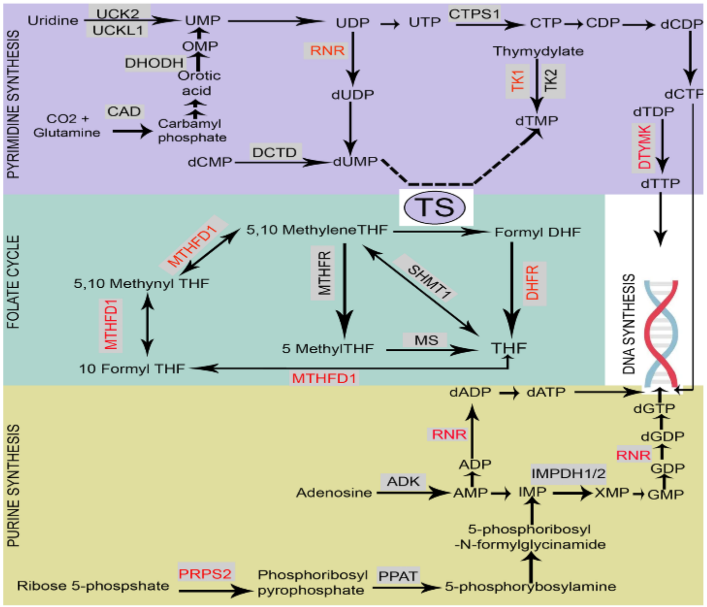

Supplemental Figure 3. Correlation between TS gene expression and nucleotide synthesis regulating genes. (A) Heat map depicting expression of TS and genes involved in pyrimidine synthesis, folate cycle and purine synthesis from 47 human PanNET samples (GSE117853, subseries GSE117851). **(B)** Genes shown in A with positive Pearson coefficient and p value < 0.05 were considered positively correlated with TS expression (marked in red). **(C)** Relationship of pyrimidine synthesis, folate cycle and purine synthesis genes with TS. Enzymes marked in red are encoded by genes positively correlated with TS as shown in B.

Supplemental Figure 4

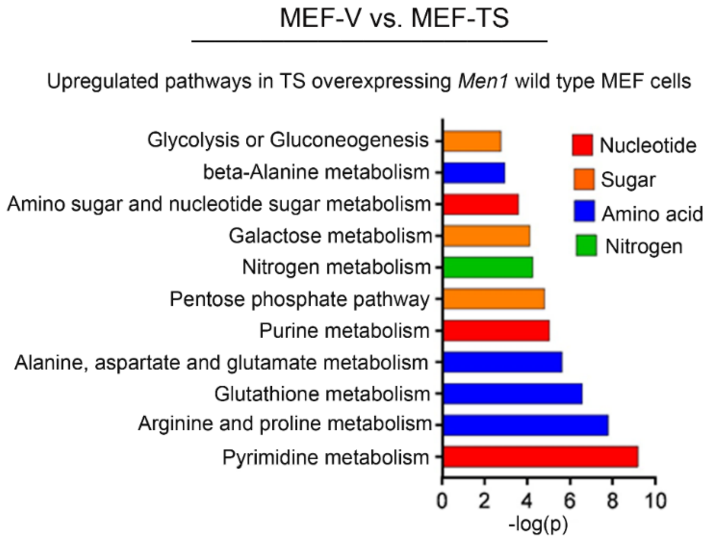

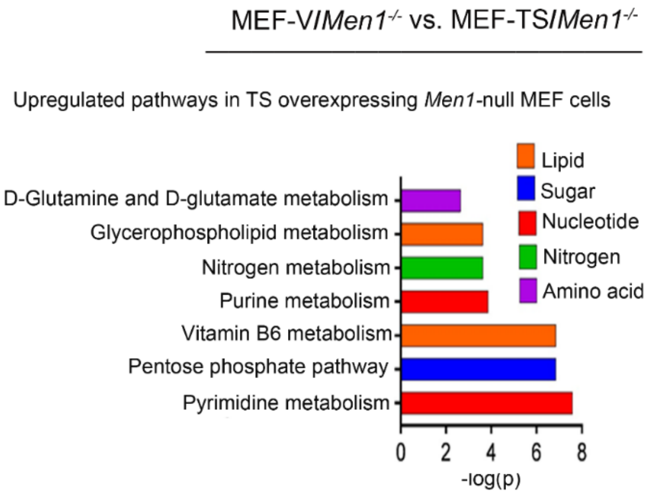

Supplemental Figure 4. KEGG pathway analysis of differentially upregulated genes involved in metabolism due to TS expression in MEF-TS and MEF-TS/*Men1*$^{-/-}$ cells vs. corresponding controls. "–log (p)" in y-axis refers to negative natural logarithmic value of the original p value from statistical analysis (pathway enrichment analysis using hypergeometric test).

Supplemental Figure 5

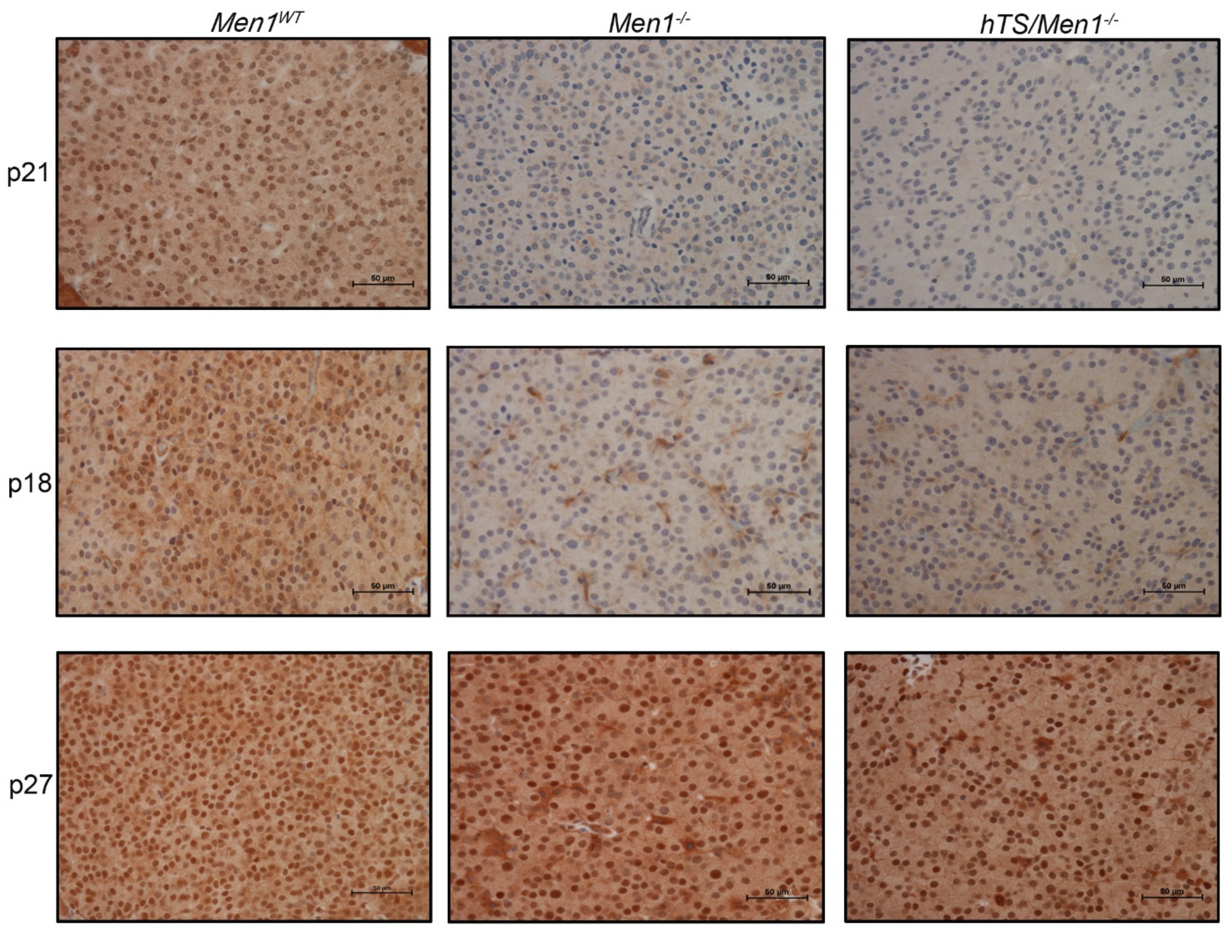

Supplemental Figure 5. Immunohistochemical staining of p21, p18 and p27 expression in *Men1*^{WT}, *Men1*$^{-/-}$ and *hTS/Men1*$^{-/-}$ mice. Scale bar, 50 µm.

Supplemental Figure 6

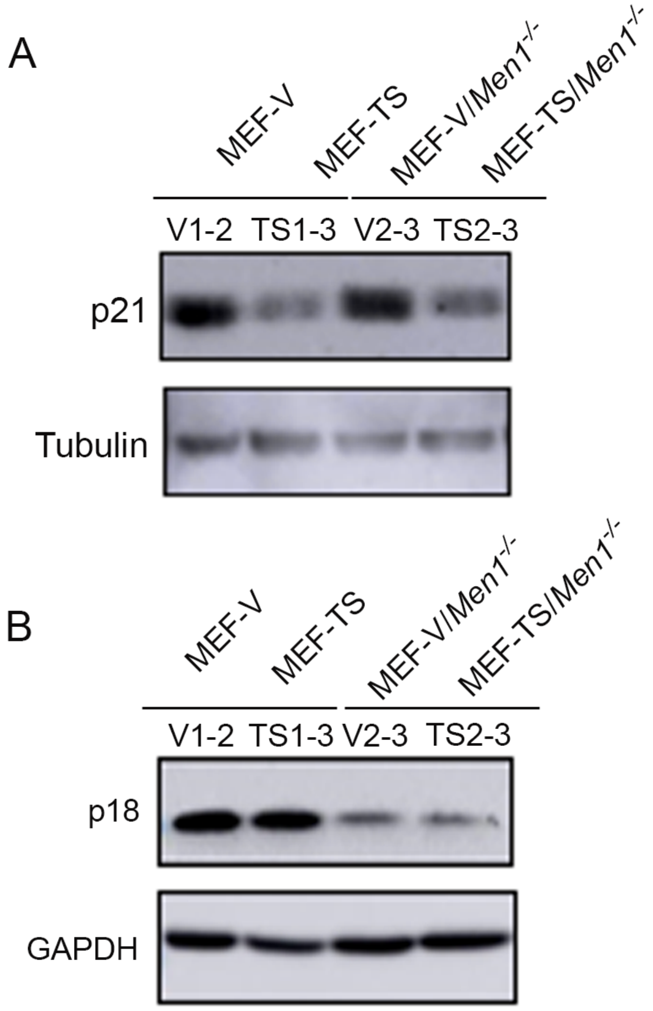

Supplemental Figure 6. Expression of p21 and p18 in MEF cell clones with overexpressed TS with and without *Men1* deletion. (A) Expression of p21 in V1-2 (MEF-V clone), TS1-3 (MEF-TS clone), V2-3 (MEF-V/*Men1*$^{-/-}$ clone), and TS2-3 (MEF-TS/*Men1*$^{-/-}$ clone). The expression of p21 in TS1-3 and TS2-3 clones was reduced when compared to control V1-2 and V2-3 clones. Tubulin was used as loading control. **(B)** Expression of p18 in V1-2 (MEF-V clone), TS1-3 (MEF-TS clone), V2-3 (MEF-V/*Men1*$^{-/-}$ clone), and TS2-3 (MEF-TS/*Men1*$^{-/-}$ clone). The expression of p18 was reduced in *Men1* null cells as compared to MEF clones with wild type *Men1.* GAPDH was used as loading control.

Supplemental Figure 7

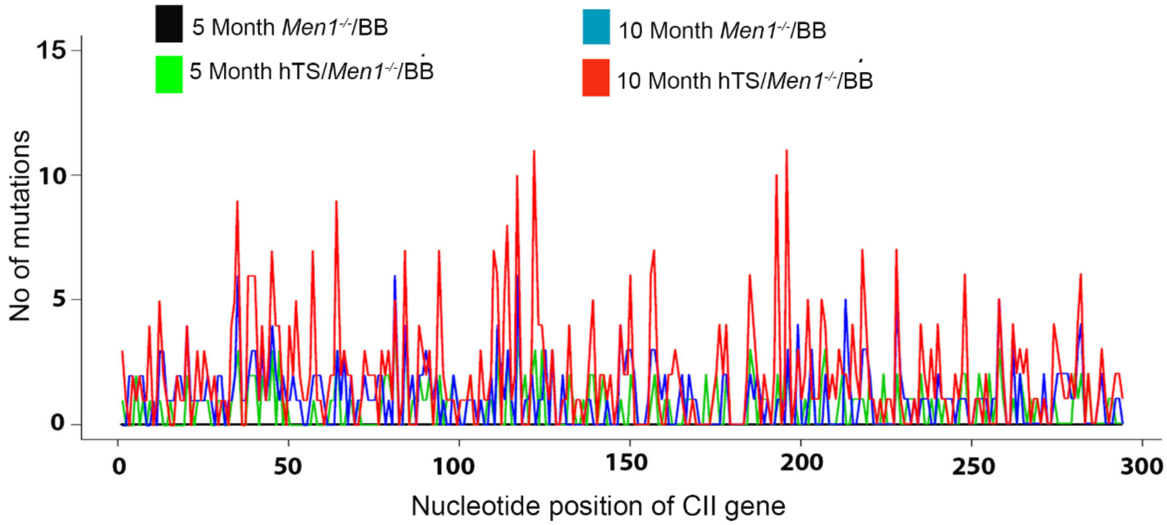

Supplemental Figure 7. Mutational spectra of CII genes recovered from *hTS/Men1*$^{-/-}$/BB and *Men1*$^{-/-}$/BB mice. Mutational spectra of CII genes recovered from 5- and 10-month-old *hTS/Men1*$^{-/-}$/BB and *Men1*$^{-/-}$/BB mice showing number of mutations in different nucleotide positions of CII genes due to hTS overexpression.

Supplemental Figure 8

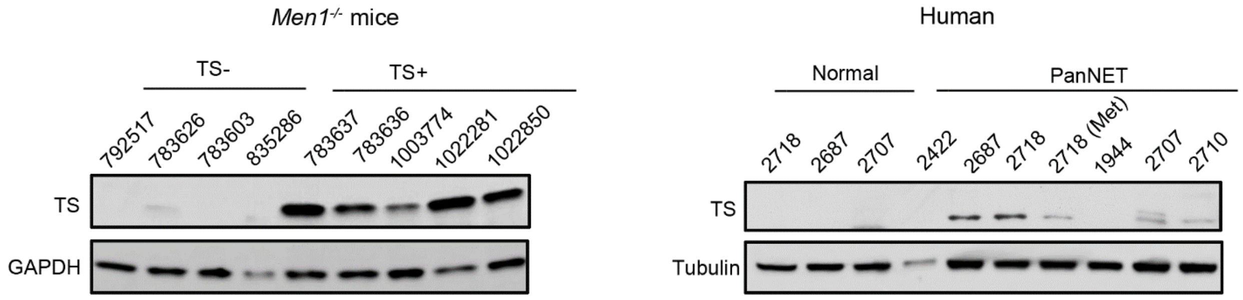

Supplemental Figure 8. Expression of TS in mouse and human PanNET tissues. (A) Expression of TS in PanNET isolated from *Men1*$^{-/-}$ (TS-) and *hTS*/*Men1*$^{-/-}$ (TS+) mice (7.5 and 10.5-month-old). GAPDH was used as loading control. Numbers above the lanes correspond to mouse ID number. **(B)** Expression of TS in pancreatic tumor isolated from human PanNET tissues. Tubulin was used as loading control. Numbers shown above the lanes correspond to patient ID number.

Supplemental Figure 9

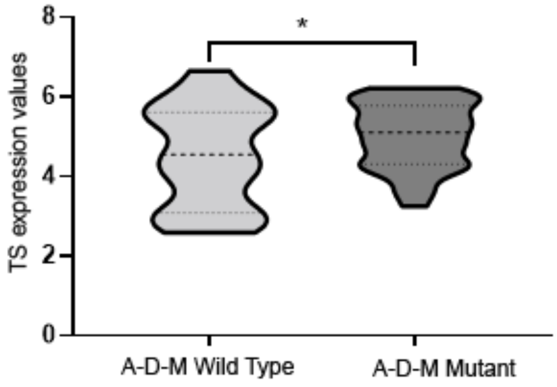

Supplemental Figure 9. TS expression in human PanNET samples. Comparison of TS expression in PanNET samples with mutations in *ATRX*, *DAXX*, or *MEN1* genes (designated A-D-M mutant PanNETs, n = 30) and in PanNET samples carrying wild type alleles of all three genes (designated A-D-M Wild Type PanNETs, n = 17); (* p = 0.044). Data obtained from GSE117853 (subseries: GSE117851). Significance calculated by one-tailed Student's t-test.

Supplemental Figure 10

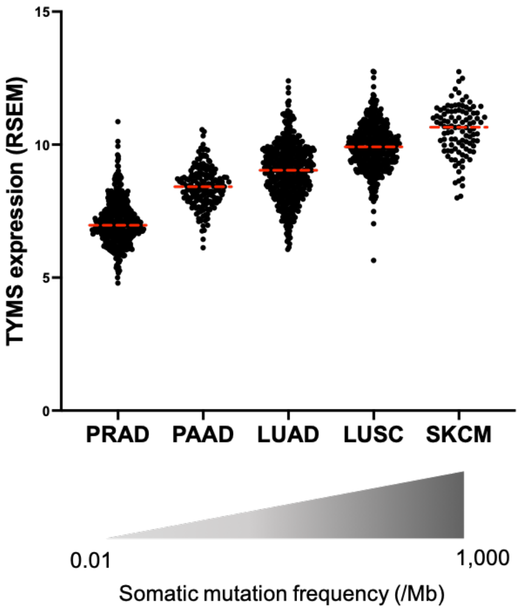

Supplemental Figure 10. Increase of TS expression is associated with increase of somatic mutations frequency. Violin graph showing the relationship between TS expression values and somatic mutation frequency in distinct tumor types. Each dot corresponds to TS mRNA levels of one patient. Tumor types are organized by increasing median TS expression levels and somatic mutation frequency. PRAD: prostate adenocarcinoma, n = 497; PAAD: pancreatic adenocarcinoma, n = 178; LUAD: lung adenocarcinoma, n = 515, LUSC: lung squamous cell carcinoma, n = 501; and SKCM: skin cutaneous melanoma, n = 103. TS expression data was obtained from TCGA and CPTAC tumor collection (http://www.linkedomics.org); somatic mutations data from Lawrence *et al.*

Supplemental Table 1. **Incidence of pancreatic islet lesions in *Men1*$^{-/-}$ vs. *hTS/Men1*$^{-/-}$ mice sacrificed at 5, 6.5 and 8 months during PanNET progression.**

Age (months)	Pancreatic Islet Lesions	*Men1*$^{-/-}$		*hTS/Men1*$^{-/-}$	
		n=16	(%)	n=16	(%)
5	Normal	1	6.25	0	0
	Hyperplasia	15	93.8	13	81.3
	Adenoma	0	0	3	18.8
	Carcinoma	0	0	0	0
6.5	Normal	0	0	0	0
	Hyperplasia	11	68.8	7	43.8
	Adenoma	5	31.3	4	25
	Carcinoma	0	0	5	31.3
8	Normal	0	0	0	0
	Hyperplasia	6	37.5	2	12.5
	Adenoma	5	31.3	5	31.3
	Carcinoma	5	31.3	9	56.3

Supplemental Table 2. Incidence of pancreatic islet lesions in *Men1*$^{-/-}$ vs. *hTS/Men1*$^{-/-}$ mice from Supplemental Table 1 combined with mice sacrificed at endpoint.

Age (months)	Pancreatic Islet Lesions	*Men1*$^{-/-}$		*hTS/Men1*$^{-/-}$	
		# of mice with tumors/ total # of mice	%	# of mice with tumors/ total # of mice	%
< 8	Normal	1/20	5	0/23	0
	Hyperplasia	15/20	75	10/23	43.5
	Adenoma	4/20	20	8/23	34.8
	Carcinoma	0/20	0	5/23	21.7
8 ~ 10	Normal	0/15	0	0/15	0
	Hyperplasia	4/15	26.7	0/15	0
	Adenoma	7/15	46.7	7/15	46.7
	Carcinoma	4/15	26.7	8/15	53.3
≥ 10	Normal	0/32	0	0/18	0
	Hyperplasia	0/32	0	0/18	0
	Adenoma	10/32	31.3	2/18	11.1
	Carcinoma	22/32	68.8	16/18	88.9

Supplemental Table 3. Pancreatic islet lesions in *Men1*$^{+/\Delta N3\text{-}8}$ and *hTS*/*Men1*$^{+/\Delta N3\text{-}8}$ mice at different stages of PanNET progression.

Age (months)	Adenoma				Carcinoma			
	hTS (-)		hTS (+)		hTS (-)		hTS (+)	
	# of mice with tumors/total # of mice	(%)	# of mice with tumors/total # of mice	(%)	# of mice with tumors/total # of mice	(%)	# of mice with tumors/total # of mice	
< 9	2/16	12.5	0/15	0	0/16	0	2/15	13.3
9~14	4/17	23.5	14/50	28.0	0/17	0	12/50	24
15~29	2/8	25.0	16/63	25.4	0/8	0	37/63	58.7

Supplemental Table 4. **SKY analysis of chromosomal abnormalities in hTS-transfected MEF/*Men1*$^{-/-}$ cells and their corresponding vector transfected controls.**

Cell type	Clones	Cell no	Karyotype
MEF-V/*Men1*$^{-/-}$	V2-1	1	43, XYY, T(6;19), +10
		2	41, X, T(6;19), +10,
		3	41, XY, T(6;19),+10
		4	41, XY, T(6;19), -13
		5	41, X, T(6;19), +10,
		6	41, XY, T(6;19), -6,+10
		7	42, XY, T(6;19), +10
		8	42, XY, T(6;19), +10
		9	43, XYY, T(6;19), +10
		10	41, XY, T(6;19), +10, -19
	V2-2	1	77, XXY, -1,-3, Der(6) T(6;19), +10,-13,-16,
		2	80, XXY, -2, -3, T(5;3), Der(6) T(6;19), +10
		3	82, XXY, Der(6) T(6;19), +10, i13, -19
		4	78,XX, -3, -5, -7, -14, -18, +10, i13
		5	80, XXY, -3, -5, -7, T(6;19), +10, i13
		6	81, XXY, Der(6) T(6;19), +10, i13
		7	78, XYYYY, -1, -2, -5, Der6 T(6;19), +10, -14,-18, i13
		8	77, XXYY, -1,-3, Der6 T(6:19), -8, +10, -19
		9	56 XXYY, -1, -2, -3, -4, -5, 6, -7, -8, -9, -11, -12, -13, -14, -15, -16, -17, -18, -19, +10
		10	79, XXY, -2, -3,-4, -5,Der6 T(6;19), +10, i13
	V2-3	1	43, XYY, T(6;19), +3
		2	41, X, T(6;19), +6
		3	41, XY, T(6;19),-13
		4	41, XY, T(6;19), -13
		5	41, X, T(6;19), +10,
		6	41, XY, T(6;19), -2
		7	40, XY, T(6;19)
		8	40, XY, T(6;19)
		9	43, XYY, T(6;19)
		10	41, XY, T(6;19), +10, -19,+12
	V2-4	1	43, XYY, T(6;19), +2
		2	41, X, T(6;19)
		3	40, XY, T(6;9)
		4	40, XY, T(6;9)
		5	39, X, T(6;19)
		6	40, XY, T(6;19), +10, -13
		7	39, XY, T(6;19), -13
		8	40, XY, T(6;19)
		9	43, XYY, T(6;19), +10
		10	39, XY, T(6;19), -5

Supplemental Table 4, continuation

		1	48,XXY,+Der(6)(6;19)
		2	78,XXYY,-3,-4,+Der(6)(6;19)x2, -7,+10x2,-14,-15,+17,-19
		3	82,XXYY,+Der(6)(6;19)x2, T(10;12),i(13)
		4	83,XXYY,+4,+Der(6)(6;19)x2, -7,+10x2,+Del(12),-13,-14,-16,+17,-19
	TS2-1	5	83,XXYYY,+Der(6)(6;19)x2,i(13).+T(13;19)
		6	83,XXYYY,+Der(6)(6;19)x2, T(10;12),i(13)
		7	83,XXYYY,-2,-3,-4,+Der(6)(6;19)x2,T(10;13),+10,+ i(13),14
		8	83,XXYYY,-2,-4,+Der(6)(6;19)x2,+7,+10,+T(10;12), +12,+i(13),-14
		9	83,XXYYYY,+Der(6)(6;19)x2, -7,-14,-18
		10	85,XXYYYYY,+6,+Der(6)(6;19)x2,-8,+10,-11,-12,-13
		1	41,XY, +T(6;19),+10,i(13)
		2	41,XYY,T(5;3),+T(6;19),+10,i(13),-13,-18
		3	42,XY, T(5;3),+T(6;19),+10,i(13)
		4	42,XYY, T(5;3),+T(6;19),-7,+10,-13
	TS2-2	5	42,XYY,+T(6;19),+10,i(13).-16
		6	42,XYY,T(5;3),+T(6;19),-7,-9,+10,T(18;13)
		7	43,XYY, T(5;3),+T(6;19),+10, i(13)
		8	44,XXYY, T(5;3),+T(6;19),+8
		9	45,XYY,+3,+T(6;19),+10, T(19;13)
MEF-TS/*Men1* $^{-/-}$		10	45,XYYY,T(5;3),++6,T(6;19),+10,i(13)
		1	41,XY, +T(6;19),+10,i(13)
		2	41,XYY,T(5;3),+T(6;19),+10,i(13),-13,-18
		3	42,XY, T(5;3),+T(6;19),+10,i(13)
		4	42,XYY, T(5;3),+T(6;19),-7,+10,-13
	TS2-3	5	42,XYY,+T(6;19),+10,i(13).-8,+9,-11,-12,-13
		6	42,XYY,T(5;3),+T(6;19),-7,-9,+9,T(18;13)
		7	43,XYY, T(5;3),+T(6;19),+10,i(13)
		8	44,XXYY, T(5;3),+T(6;19),+8
		9	45,XYY,+3,+T(6;19),+10, T(19;13)
		10	45,XYYY,T(5;3),++6,T(6;19),+10,i(13)
		1	41,XY, +T(6;19),+10,i(13),-13,-18
		2	41,XYY,+T(6;19),+10,i(13)
		3	42,XY, T(5;3),+T(6;19),+10,i(13),-7
		4	42,XYY,+T(6;19),+10,-13
	TS2-4	5	43,XYY, T(5;3),+T(6;19),+10,i(13)
		6	44,XXYY, T(5;3),+T(6;19),+8
		7	45,XYY,+3,+T(6;19),+7
		8	42,XY,T(5;3),++6,T(6;19),+10
		9	42,XYY,+T(6;19),+10,-16
		10	42,XYY,T(5;3),+T(6;19),-7,-9,+10,T(18;13)

Supplemental Table 5. Chromosomal abnormalities in MEF-TS/*Men1*$^{-/-}$ and MEF-V/*Men1*$^{-/-}$ cells. Data quantification derived from Supplemental Table 4.

Cell lines		Chromosomal Abnormality (%)			
		Aneuploid cells	Translocation	Loss	Gain
MEF-V/*Men1*$^{-/-}$	Clone V2-1	100	0	50	90
	Clone V2-2	100	60	100	100
	Clone V2-3	70	0	40	60
	CloneV2-4	70	0	50	30
	Average	85	15	60	70
	Standard Error	7.75	13.42	12.11	14.14
MEF-TS/*Men1*$^{-/-}$	TS2-1	100	50	70	100
	TS2-2	100	100	40	100
	TS2-3	100	100	40	100
	TS2-4	100	70	50	100
	Average	100	80	50	100
	Standard Error	0.00	10.95	6.32	0.00

Supplemental Table 6. Nucleotide changes associated to hTS overexpression in 5 and 10-month-old *Men1*$^{-/-}$/BB (hTS-) and *hTS/Men1*$^{-/-}$/BB (hTS+) mice.

Type of Mutations		5 months		10 months	
		hTS(-)	hTS(+)	hTS(-)	hTS(+)
Transversions	GC>TA	0	42	45	134
	GC>CG	0	64	57	140
	AT>TA	0	35	27	82
	AT>CG	0	65	68	135
Transitions	GC>AT	0	66	77	187
	AT>GC	0	38	28	73
Insertions		0	12	7	22
Deletions		0	40	22	58
Total mutations		0	362	331	831

Supplemental Table 7. Characteristic features of clinical specimens used to study correlation between TS protein expression and patient outcome. Pearson's chi-squared test is used to analyze statistical significance. For tumor size, Linear-by-Linear association is used; ns - statistically non-significant, * $p < 0.05$.

	Total	TS expression status				p value
		TS negative samples (Out of total 70 TS negative PanNETs)		TS positive samples (Out of total 18 TS positive PanNETs)		
		No.	%	No.	%	
**Sex**						ns
Male	43	33	47.1	10	55.6	0.524
Female	45	37	52.9	8	44.4	
**Function**						ns
Non-functional	69	54	77.1	15	83.3	0.684
Hypoglycemia	9	7	10	2	11.1	
GI symptom	10	9	12.9	1	5.6	
**Syndrome**						ns
Absent	80	65	92.9	15	83.3	0.13
VHL	5	4	5.7	1	5.6	
MEN1	3	1	1.4	2	11.1	
**Location**						ns
Head	41	33	47.1	8	44.4	0.492
Body	18	15	21.4	3	16.7	
Tail	22	18	25.7	4	22.2	
Multifocal	7	4	57.1	3	16.7	
**Size**						*
< 2 cm	32	27	38.6	5	27.8	0.046
≥ 2 cm, < 5 cm	44	37	52.9	7	38.9	
≥ 5 cm	12	6	8.6	6	33.3	
**Angioinvasion**						ns
Absent	58	48	68.6	10	55.6	0.299
Present	30	22	31.4	8	44.4	
**Perineural invasion**						ns
Absent	73	58	82.9	15	83.3	0.962
Present	15	12	17.1	3	16.7	
**LN metastasis**						ns
Absent	52	40	57.1	12	66.7	0.614
Present	8	6	8.6	2	11.1	
Not evaluable	28	24	34.3	4	22.2	
**Invasion depth**						ns
Confined	63	52	74.3	11	61.1	0.269
Extension	25	18	25.7	7	38.9	
**WHO classification**						*
G1	64	52	74.3	12	66.7	0.02
G2	20	17	24.3	3	16.7	
G3	4	1	1.4	3	16.7	

Supplemental Table 8. Correlation between TS gene expression and expression of genes involved in DNA damage response. Grey background indicates genes positively regulated with TS expression.

GENE NAME	ACTIVITY LINKED TO OMIM	Correlation with TS gene expression	
**Base excision repair (BER)**	**DNA glycosylases: major altered base released**	**Pearson coefficient**	**p value**
UNG	U	0.1657	0.2655
SMUG1	U	0.3125	0.0324
MBD4	U or T opposite G at CpG sequences	-0.1752	0.2389
TDG	U, T or ethenoC opposite G	-0.1286	0.389
OGG1	8-oxoG opposite C	-0.1975	0.1832
MUTYH (MYH)	A opposite 8-oxoG	-0.4215	0.0032
NTHL1 (NTH1)	Ring-saturated or fragmented pyrimidines	-0.1232	0.4094
MPG	3-meA, ethenoA, hypoxanthine	0.2317	0.1172
NEIL1	Removes thymine glycol	-0.2346	0.1124
NEIL3	Removes oxidative products of pyrimidines	0.396	0.0059
Percentage of genes showing positive correlation with TS		20%	
**Mismatch excision repair (MMR)**			
MSH2	Mismatch (MSH2-MSH6) and loop (MSH2-MSH3) recognition	0.3151	0.031
MSH3		0.095	0.5213
MSH6		0.3417	0.0187
MLH1	MutL homologs, forming heterodimer	-0.09684	0.5173
PMS2		-0.01214	0.9355
MSH4	MutS homologs specialized for meiosis	-0.2039	0.1693
MSH5		-0.4072	0.0045
MLH3	MutL homologs of unknown function	0.0016	0.99
PMS1		-0.2378	0.1075
PMS2P3 (PMS2L3)		0.885	0.554
Percentage of genes showing positive correlation with TS		20%	
**Modulation of nucleotide pools**			
NUDT1 (MTH1)	8-oxoGTPase	0.6508	<0.0001
DUT	dUTPase	0.1289	0.3879
PARK7 (DJ-1)	Guanine glycation repair	-0.1537	0.3022
DNPH1	Hydrolase for 5-hydroxymethyl deoxyuridine	0.3179	0.0294
NUDT15 (MTH2)	Hydrolysis of thiopurines?	0.4775	0.0007
NUDT18 (MTH3)	Hydrolysis of 8-hydroxypurine diphosphates	0.1389	0.3517
Percentage of genes showing positive correlation with TS		50%	
**Homologous recombination**			
RAD51	Homologous pairing	0.4156	0.0037
RAD51B	Rad51 homolog	-0.29	0.044
RAD51D	Rad51 homolog	0.071	0.63
SPIDR	Shu subunits, RAD51 recruitment	0.06418	0.6682
PDS5B		-0.033	0.02
DMC1	Rad51 homolog, meiosis	-0.257	0.08
XRCC2	DNA break and crosslink repair	-0.4449	0.0017
XRCC3		-0.1063	0.4769
RAD52	Accessory factors for recombination	-0.374	0.0095
RAD54L		0.1266	0.3964
RAD54B		0.3149	0.0311
BRCA1	Accessory factor for transcription and recombination, E3 Ubiquitin ligase	0.0307	0.8377
BARD1	BRCA1-associated	0.2866	0.0508
PAXIP1 (PTIP)	MDC1 paralog in 53BP1 pathway	0.4785	0.0007
SMC5	Recruit cohesion during HR	-0.17	0.252
SMC6		-0.03152	0.8334
SHLD2 (FAM35A)	Suppressing end-resection	-0.1692	0.2555
SEM1 (SHFM1) (DSS1)	BRCA2 associated	0.4309	0.0025
RAD50	ATPase in complex with MRE11A, NBS1	0.3386	0.0199
MRE11A	3' exonuclease, defective in ATLD (ataxia-telangiectasia-like disorder)	0.2908	0.0474
NBN (NBS1)	Mutated in Nijmegen breakage syndrome	-0.06	0.686
RBBP8 (CtIP)	Promotes DNA end resection	0.4737	0.0008
MUS81	Subunits of structure-specific DNA nuclease	-0.06275	0.6752
Percentage of genes showing positive correlation with TS		35%	
**Non-homologous end-joining**			
XRCC6 (Ku70)	DNA end binding subunit	-0.339	0.19
XRCC5 (Ku80)	DNA end binding subunit	0.1379	0.3554
PRKDC	DNA-dependent protein kinase catalytic subunit	0.3549	0.014
LIG4	Ligase	0.1408	0.3451
XRCC4	Ligase accessory factor	0.3493	0.0161
DCLRE1C (Artemis)	Nuclease	-0.2605	0.077
NHEJ1 (XLF, Cernunnos)	End-joining factor	0.1708	0.2511
Percentage of genes showing positive correlation with TS		29%	
**Other conserved DNA damage response genes**			
ATR	ATM- and PI-3K-like essential kinase	-0.204	0.167
MDC1	Mediator of DNA damage checkpoint	-0.385	0.0074
PCNA	Sliding clamp for pol delta and pol epsilon	0.7616	<0.0001
RAD1	Subunits of PCNA-like sensor of damaged DNA	0.2985	0.0415
RAD9A		0.04464	0.7657
HUS1		0.1686	0.257
RAD17 (RAD24)	RFC-like DNA damage sensor	0.3882	0.007
CHEK1	Effector kinases	0.2005	0.066
CHEK2		0.2927	0.0459
TP53	Regulation of the cell cycle	-0.05	0.7
TP53BP1 (53BP1)	chromatin-binding checkpoint protein	-0.2811	0.055
RIF1	suppressor of 5'-end-resection	0.0271	0.8563
TOPBP1	DNA damage checkpoint control	0.2642	0.0727
CLK2	S-phase check point and biological clock protein	-0.1735	0.2435
Percentage of genes showing positive correlation with TS		28.50%	
**Chromatin Structure and Modification**			
H2AX (H2AFX)	Histone, phosphorylated after DNA damage	-0.2085	0.1597
CHAF1A (CAF1)	Chromatin assembly factor	0.1396	0.3495
SETMAR (METNASE)	DNA damage-associated histone methylase and nuclease	0.107	0.4743
ATRX	Chromatin remodeling, transcription factor	-0.1	0.4625
Percentage of genes showing positive correlation with TS		0.00%	
**Poly(ADP-ribose) polymerase (PARP) enzymes that bind to DNA**			
PARP1 (ADPRT)	Protects strand interruptions	0.1031	0.4903
PARP2 (ADPRTL2)	PARP-like enzyme	0.2512	0.0885
PARP3 (ADPRTL3)	PARP-like enzyme	-0.03158	0.8331
PARG	Poly(ADP-ribose) glycohydrolase	0.03292	8261
PARPBP	Binds PARP and modulates recombination	0.535	0.0001
Percentage of genes showing positive correlation with TS		20.00%	
**Percentage of DNA damage response genes analyzed that correlate with TS expression**		**27.80%**	

Supplemental Table 9. **Antibodies used in the study.**

Name	Catalog #	Antibody Dilution
**Antibodies for IHC in mice tissues**		
Menin	Bethyl Lab Inc, Cat# A300-105A	1:4000
p27	Thermo Fisher Scientific, Cat# MS-256-P1	1:100
p18	Abcam, Cat# ab78379	1:75
p21	Santa Cruz biotechnology, Cat# sc-397	1:500
Ki-67	Vector Lab, Cat# VP-K451	1:10,000
**Antibodies for IHC in Human tissues**		
TS	Fisher Scientific, Cat# MAB4130MI	1:100
ɣ-H2AX	Upstate, Cat# 05-636	1:300
Ki-67	Abcam, Cat# ab92742	1:500
**Antibodies for Immunoblotting**		
Menin	Bethyl lab, Cat# A300-105A	1:1000
p18	Thermo Scientific, Cat# MS-858	1:1000
p21	BD Pharmingen, Cat# 556431	1:1000
p27	Abcam, Cat# AB7961	1:1000
ɣ-H2AX	Cell signaling technology, Cat# 9718	1:1000
TS (Clone TS-106)	As previously described (14)	1:300
Anti-rabbit horseradish peroxidase-conjugated secondary antibody (used for detecting Menin, ɣ-H2AX and p27)	Bio-Rad, Cat# 1706515	1:5000
Anti-mouse horseradish peroxidase-conjugated secondary antibody (used for detecting TS, p18 and p21)	Bio-Rad, Cat# 1706516	1:5000
**Antibodies for Immunofluorescence**		
ɣ-H2AX	Novus Biologicals, Cat# NB100-79967	1:100
Alexa Fluor-conjugated secondary antibodies	Invitrogen, Cat# A11037	1:300
